# Prenatal arsenic exposure and gene expression in fetal liver, heart, lung, and placenta

**DOI:** 10.1016/j.tox.2026.154420

**Published:** 2026-02-10

**Authors:** Kristal A. Rychlik, Sylvia S. Sanchez, Chloe Kashiwagi, Jin-Shiung Liao, Aakriti Mathur, Emily J. Illingworth, Andre Kleensang, Alexandra Maertens, Fenna C.M. Sillé

**Affiliations:** a Department of Environmental Health and Engineering, Bloomberg School of Public Health, Johns Hopkins University, Baltimore, MD, USA; b Public Health Program, School of Health Professions, Mayborn College of Health Sciences, University of Mary Hardin-Baylor, Belton, TX, USA

**Keywords:** Arsenic, Prenatal, MRNA, Microarray, Immune

## Abstract

Prenatal arsenic exposure has been linked to a myriad of negative health effects. There is relatively little insight into the mechanisms and signaling alterations across different fetal organs that drive long-term immune-related issues following prenatal arsenic exposure. Therefore, the effects of this exposure window on gene expression in the liver, placenta, heart, and lung of gestation day (GD) 18 C57BL/6 mouse fetuses were investigated. From two weeks prior to mating until tissue collection at GD18, mice were exposed to 0 or 100 ppb sodium (meta) arsenite in drinking water, *ad libitum*. Genes of interest were analyzed by RT-qPCR, complemented with untargeted Agilent 44 K microarray analysis. Data cleanup and analysis was performed in RStudio. Differentially expressed mRNAs were queried in the String Database and using Cytoscape to create interaction networks and identify significantly enriched biological pathways. A total of 251, 165, 158, and 41 genes were significantly altered in the liver, placenta, heart, and lung, respectively, when treated samples were compared to controls. Many altered pathways were immune-related, supporting prior research. Most notably, gene expression of *Gbp3*, a key player in the cellular response to interferon gamma, was found to be reduced in placentas of male fetuses exposed to arsenic compared to controls (*p* = 0.0237).

**Impact:**

This is the first study comparing alterations in gene expression across multiple organs following prenatal exposure to environmentally relevant levels of arsenic. These findings, elucidating the multi-organ impact of prenatal arsenic exposure on predominantly immune-related pathways, further our mechanistic understanding of the long-term health effects observed in early-life arsenic-exposed populations.

## 1. Introduction

Despite strong population-level data supporting the association between prenatal inorganic arsenic (iAs) exposure and cancer of the liver and lung, heart disease, and immune dysfunction (Smith et al. 2012; M. F. Naujokas et al. 2013; Khan et al. 2020), the mechanisms driving these long-term effects are still unclear. Arsenic is the most common chemical drinking water contaminant worldwide with approximately 230 million people at risk (Shaji et al. 2021), including the most vulnerable populations of pregnant women and children. In the U.S. alone, an estimated 2.1 million people are drinking water contaminated with levels of arsenic higher than the recommended limit of 10 parts per billion (ppb) (Ayotte et al. 2017). As reviewed by Naujokas et al. (2013), levels of arsenic around the world range widely, from below the U.S. Environmental Protection Agency (US EPA) and World Health Organization (WHO) standards of 10 ppb to the thousands of ppb (Marisa F. Naujokas et al. 2013). For example, García Salcedo et al. (2022) asserts that pregnant mothers in Comarca Lagunera, Mexico were exposed to average levels of 47.7 μg/L as tested in various biological samples (García Salcedo et al. 2022). Similarly, increased levels of prenatal arsenic exposure, up to 262 μg/L, were associated with older age of menarche in girls in Bangladesh (Rahman et al. 2021). Therefore, 100 ppb is an environmentally and biologically relevant dose for many populations across the globe from Mexico, the US and Chile, to Ghana, China, Bangladesh and Vietnam (Guan et al. 2012; Naujokas et al. 2013; Navasumrit et al. 2019; Chen and Costa, 2021; García Salcedo et al. 2022).

In an effort to isolate the mechanisms driving effects, recent studies have begun to investigate the placenta. Identified as a route of early exposure to arsenic (García Salcedo et al. 2022), the placenta is a key mediator of *in utero* exposure and, in the case of heavy metals, may provide an indication of altered signaling in the neonate due to maternal exposure (Myllynen et al. 2005). Early evidence, albeit at rather high doses (20 ppm), indicate disruption of placental vasculogenesis (He et al. 2007) which could result in placental insufficiency, a condition which, like arsenic, has been associated with fetal (or intrauterine) growth restriction (IUGR) (Neerhof and Thaete, 2008; Xu et al. 2022).

Several biological pathways have been explored as potential drivers of adverse prenatal outcomes. One heavily studied pathway that has been presented as a possible mediator for IUGR after prenatal arsenic exposure is the glucocorticoid receptor signaling pathway (Caldwell et al. 2015). Not only is this pathway essential for many endocrine functions but it also plays a key role in metabolism and immune function (Kadmiel and Cidlowski, 2013). Dose-dependent effects on glucocorticoid signaling has also been identified in JEG-3 cells, an *in vitro* model of trophoblasts (Meakin et al. 2019). Specifically, Meakin et al. (2019) identified DNA methylation as a key mechanism driving these effects. Epigenetic alterations and gene expression changes have also been identified in newborn mouse liver tissue following *in utero* exposure to 85 ppm arsenic (Xie et al. 2007). Therefore, glucocorticoid signaling can influence immune response and downstream developmental outcomes either directly or indirectly.

Disruption of immune signaling has been identified as a major pathway through which prenatal arsenic exposure can impact sex differences, gene expression changes, epigenetic programming and other outcomes. For instance, in a U.S. cohort study of low-to-moderate levels of arsenic exposure during pregnancy, alterations in gene expression and the epigenetic profile of placental tissue were observed with differences noted between male and female fetal sex (Winterbottom, Ban, et al. 2019; Winterbottom, Moroishi, et al. 2019). In human cohorts, gene expression and cytokine levels have been affected in cord blood based on maternal arsenic exposure levels (Fry et al. 2007; Rager et al. 2014). Furthermore, in an integrative study of data from twelve prior cohort studies in humans looking at CpG methylation, gene expression, or protein expression in the placenta or cord blood, Laine & Fry (2016) determined that many of the commonly altered genes were related to immune and inflammatory pathways. Lastly, arsenic induced renal toxicity and neurobehavioral changes have been consistently linked to alterations in the oxidative stress pathways and signaling mechanisms across multiple species (Xi et al. 2010; Lu et al. 2024; Singh et al. 2025).

Despite these findings, it remains unclear whether gene expression alterations observed in the placenta at birth correspond to alterations in other organs and whether those alterations are mechanistic drivers for long-term disease. Therefore, this study aimed to determine what gene expression alterations, if any, occurred following preconception and prenatal exposure to inorganic arsenic in drinking water among placenta, liver, heart, and lung tissues in the C57Bl/6 J mouse model. This animal model is well characterized (Song and Hwang, 2017; Genome Reference Consortium, 2020) and human analogues have been identified for many genes involved in metabolism, the immune system (Rydell-Törmänen and Johnson, 2019), and various other pathways shown to be affected by arsenic exposure. Physiologic effects of prenatal exposure have been previously characterized in this model (Rychlik et al. 2023) and neurodegenerative and metabolic effects observed in chronically exposed human populations are also conserved in this model (Castriota et al. 2020; Lai et al. 2024; Zhang et al. 2025). Genes of interest were selected for quantification using RT-PCR in each organ based on prior research and biological networks of interest, particularly those at the intersection between the immune system and glucocorticoid signaling. In addition, a microarray analysis was performed on a subset of samples to assess larger network alterations and trends.

## 2. Materials and methods

### 2.1. Animal care and mouse exposure model

**Fig. 1.**
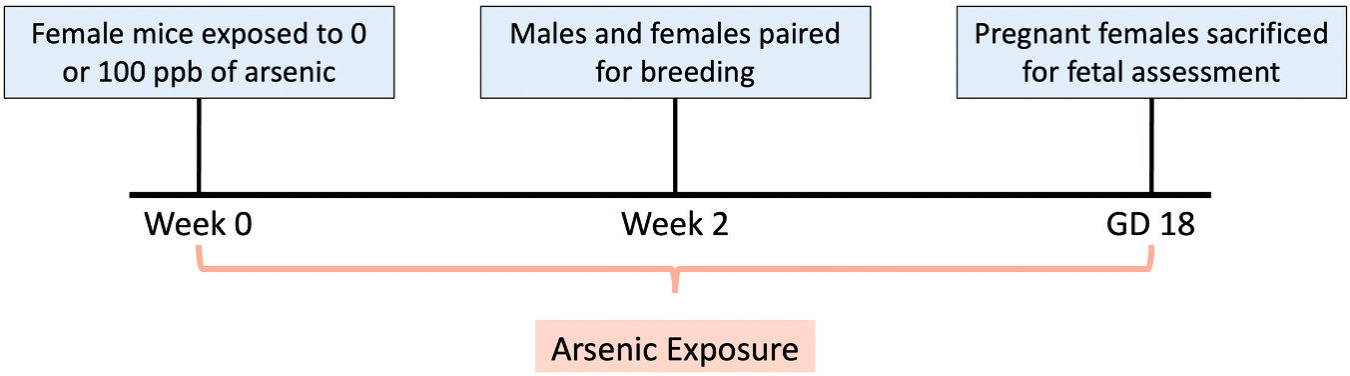
Preconception and prenatal arsenic exposure paradigm. 23 C57Bl/6 J mice were exposed to either 0 ppb (control) or 100 ppb (exposed) sodium (meta) arsenite (iAs) in drinking water from two weeks prior to breeding until euthanasia of the pregnant dam at gestational day (GD) 18. Samples from 3 to 6 randomly selected fetuses with matching placentas, each from a unique litter, were collected at GD 18 including liver, heart, lung, and placenta and flash frozen using liquid nitrogen.

Despite some interspecies differences, C57BL/6 mouse models have been proven representative for assessing arsenic immunotoxicity (Milton and Rahman, 2002; Kozul et al. 2009; Ramsey, Larcombe, et al. 2013; Ramsey, Bosco, et al. 2013; Li et al. 2017; Wu et al. 2017; Castriota et al. 2020). Breeder male and female C57Bl/6 J mice (8 weeks of age) were obtained from Jackson Laboratories (Bar Harbor, Maine). Mice were given a week to acclimate prior to conducting any experiments. Male mice were housed individually. Female mice were housed in groups of five prior to mating, after which they were housed individually in order to accurately monitor food and water intake. Enrichment was added to cages including a mouse igloo and additional sterile paper bedding material. Cages were housed in a temperature and humidity-controlled facility with 14:10 light:dark cycle and provided *ad libitum* access to food and water. Mice were fed low arsenic chow (AIN-93M: Research Diets, New Brunswick, NJ, CAT# D10012Mi). **Exposure model:** Mice were evenly distributed (by weight) into two groups (10 breeding pairs per group) two weeks prior to mating: Control with *ad libitum* access to arsenic-free water (0 ppb iAs) and Exposure with access to exposure water (spiked with 100 ppb iAs - a relevant dose in many areas of the world (Naujokas et al. 2013; Chen and Costa, 2021; García Salcedo et al. 2022) - in the form of sodium(meta)arsenite (CAS# 7784-46-5) obtained from Millipore Sigma [St. Louis, MO]). This dose represents a relatively low, but common dose in the global context even though it is higher than the US EPA and WHO standards (Naujokas et al. 2013; Chen and Costa, 2021; García Salcedo et al. 2022). All water was prepared using Crystal Springs brand water (Lakeland, FL), with reported non-detectable levels of arsenic, and was refreshed every 3–4 days to avoid arsenic oxidation. Water was provided to the mice in glass containers to avoid risk of phytoestrogen interference in the study. The exposure paradigm is represented in Fig. 1. **Breeding strategy:** To obtain fetuses, timed matings were carried out by exchanging female and male bedding three days prior to mating, and then pairing 1:1 male: female in the male cage overnight. The next morning was designated GD0.5 (gestational day 0.5) and males were removed to avoid asynchronous pregnancies. Successful pregnancy was confirmed by weight gain. Of the 10 breeding pairs per group, only 5 (controls) and 6 (treatment) dams achieved pregnancy. Fetuses were harvested from the first litter of pregnant females euthanized by CO_2_ overdose on gestational day (GD) 18, resulting in samples from 11 total breeding pairs. This time point was selected based on evidence that GD18 represents a late gestation stage in which major organ systems, including the immune and respiratory systems, experience rapid maturation and major shifts in gene expression (Besnard et al. 2011; Paquette et al. 2018; Moore et al. 2022; Li et al. 2025).

Placenta, lung, liver, and heart tissue were collected from fetuses and flash frozen using liquid nitrogen. **Sample size:** For the microarray experiment 2 different exposure groups (unexposed vs. 100 ppb inorganic arsenic exposed) * 4 different tissues (fetal heart, liver, lung and placenta) * 2–3 animals per exposure group were used. These numbers reflect a total of 3 breeding pairs and 6 pups used for the microarray experiment, with each biological replicate representing a unique litter. For the validation by RT-qPCR the remaining fetal tissue samples (including those from littermates to increase total n) were used, and the number of samples varied across tissue type and gene analysis due to fetal sample availability (Supplemental Table 1). Based on prior literature in other comparable studies (Torres et al. 2016; Vilmundarson et al. 2022), biological triplicates (n = 3) for each tissue per exposure group was determined to be sufficient for the current microarray analysis as the observed transcriptomic changes were further paired with RT-qPCR validation. **Randomization:** Each dam was assigned a number and, using a random number generator (https://www.random.org/), was assigned to either control or treatment. If, after randomization, it was found that the weights of the animals within each treatment arm were not matched, further rounds of randomization were conducted until animals were roughly matched in weight between the treatment arms. Upon sacrifice, a random fetus and matching placenta was chosen from each litter (n = 3 per exposure group) for RNA extraction, using the same random number generator tool. Sex as a biological variable was not determined nor considered during the randomization process for fetal collection. Though not the primary objective of this paper due to the uncertainty of the number of pups and sex in each litter, when sample size and study design permitted, analyses were stratified by fetal sex to explore sex specific effects. Male cages were placed lateral, above and below the female cages which were placed in an alternating sequence (control, treatment, control, etc.) in the central area of a housing rack to minimize noise and vibration disturbance stressors. **Inclusion & exclusion criteria:** Only pregnant females were included in this study to obtain access to fetal tissues. Tissue samples were excluded from analysis if contamination occurred, if RNA yield was less than the 100 ng required for the microarray, or if RNA quality was poor (pure was considered 260/280 ratio >2.0; 2.0 <260/230 ratio< 2.2; and 260/230 ratio > 260/280 ratio). One sample was excluded from the microarray analysis after not passing post-run quality control. For the RT-qPCR analysis, any sample exceeding 2 standard deviations from the mean was excluded. **Blinding/Masking:** During allocation of treatment groups, the postdoc conducting the study assigned treatment groups. The research assistant was responsible for weighing animals, food, and water, and dosing animals throughout the experiment period. At the time of euthanasia, treatment status was blinded for experimenters who collected biological samples. Tissues were sent for microarray analysis without identifying information about treatment and once results were obtained, the data was analyzed prior to unmasking the treatment arms using previously assigned identifiers. **Outcome measures**: For phenotypic characterization, maternal weight, food and water intake, and fetal total weight, placenta weight, and liver weight were measured. Total RNA was measured for each sample and individual gene expression was measured as detailed below.

#### 2.1.1. ARRIVE statement

This study was conducted with approval by the Johns Hopkins University Institutional Animal Care and Use Committee (Protocol # MO20H283), following the National Research Council’s Guide for the Care and Use of Laboratory Animals. All animals were housed in the Johns Hopkins School of Public Health vivarium, which is in compliance with the Animal Welfare Act regulations and Public Health Service (PHS) Policy (PHS Animal Welfare Assurance #: D16–00173 (A3272–01)), and is accredited by the Association for the Assessment and Accreditation of Laboratory Animal Care (AAALAC) International (# 000503). In addition, we have been following the ARRIVE guidelines 2.0 (Percie du Sert et al. 2020) for the entire study.

### 2.2. Total RNA isolation

Tissues were thawed in RNAprotect (Qiagen Inc., Valencia, CA), ground using frosted slides and run through a Qiashredder column (Qiagen Inc., Valencia, CA) before RNA isolation. Total RNA was extracted from each organ (<30 mg) using RNeasy Mini kits (Qiagen Inc., Valencia, CA). Quantity and purity of total RNA was measured using a Take3 Microvolume Plate and the Take3 app (Agilent Technologies, Santa Clara, CA). Total RNA was considered pure when 260/280 ratio > 2.0; 2.0 < 260/230 ratio< 2.2; and 260/230 ratio > 260/280 ratio.

### 2.3. Microarray

Total RNA was reverse-transcribed to cDNA using the Applied Bio-systems High-Capacity cDNA Reverse Transcription Kit (CAT# 4374967; Applied Biosystems, Waltham, MA).

Gene expression analysis was carried out using Agilent Whole Mouse 44 K Genome Oligo Array (Agilent Technologies, Santa Clara, CA) according to manufacturer instructions. Due to low RNA isolation yields from these small fetal tissues and quality control issues, the total number of samples included in the final analysis was n = 2–3 litter representatives per group (arsenic & control). The microarray was conducted using a smaller sample size that did not allow for sex stratification. However, the sex differences were evaluated in our downstream analysis using RT-qPCR. Although small, similar sample sizes have been widely used in exploratory developmental and toxicogenomics studies. For example, the National Toxicology Program has used a n = 3 per group for pathway level microarray analyses in short term exposure studies, which was considered sufficient for broad transcriptomic changes when paired with follow-up validation (as reviewed in (Krewski et al. 2020). Our study also couples our initial transcriptomic screening with targeted downstream validation.

### 2.4. RT-qPCR

RNA was reverse transcribed according to the manufacturer’s instructions using the high-capacity cDNA reverse transcription kit with RNase inhibitor from Applied Biosystems (CAT# 4374967; Applied Biosystems, Waltham, MA). RT-qPCR was run on a set of samples (n = 2–15 per group, from 2 to 6 different litters per group – see Supplemental Table 1) using Universal SYBR Green Fast qPCR Mix (Cat no. RK21203; ABClonal Inc., Woburn, MA) and primer sets from published literature that exhibited successful RT-qPCR results (Table 1). When determining which genes to run using RT-qPCR, a literature search was conducted using google scholar and PubMed to search for articles that investigated alterations in transcription of genes following prenatal and early life arsenic exposure. Studies that found alterations in human populations were prioritized in terms of importance and genes that seemed less well studied or more novel were selected for further analysis. All runs were normalized to the 18S ribosomal housekeeping gene (Table 1) as described previously (Espíndola et al. 2012). Taqman probes (Applied Biosystems, Waltham, MA) were utilized for proteasome 20S subunit beta 8 (*Psmb8*, CAT# 4453320), interferon regulatory factor 1 (*Irf1*, CAT# 4331182), interferon regulatory factor 9 (*Irf9*, CAT#. 4331182), and protein tyrosine phosphatase, non-receptor type 2 (*Ptpn2*, CAT# 4331182), following instructions from the manufacturer.

### 2.5. Statistical analysis

Potential differences in food and water intake as well as dam weight were determined by average group comparisons and Student *t*-test. RT-qPCR results were analyzed using GraphPad Prism 9 (GraphPad Software, Boston, MA). Groups were compared using an ANOVA with post hoc analysis Bartlett’s statistic (corrected; for Guanylate-Binding Protein 3) or Tukey’s multiple comparisons test (for all others). Significance was considered at *p* < 0.05.

Data from microarray experiments were preprocessed using Gene-Spring (Agilent Technologies, Santa Clara, CA) software. Raw data were imported and quantile-normalized. Statistical analysis was done using limma with a moderated Bayes *t*-test and a Benjamini-Hochberg correction. Although no individual gene was statistically significantly changed after correcting for multiple hypothesis testing, all genes with an unadjusted *p*-value less than 0.01 were investigated for further pathway analysis. This exploratory approach was used to identify effects across functionally related genes that could reveal biologically meaningful signals that would be missed with more stringent thresholds and single gene tests. The resulting genes would then be further validated with network analysis and RT-qPCR methods. Functional assays were not a primary objective of this study, but this approach also provided insights into downstream analyses as described previously (Khatri et al. 2012).

### 2.6. Protein-protein interaction networks and pathway enrichment

We queried over-represented pathways using Search Tool for the Retrieval of Interacting Genes/Proteins (STRING, v12.0, https://string-db.org), a database that extracts experimental and curated data from several sources to deduce protein-protein interaction (PPI) networks from a set of protein-coding genes (Szklarczyk et al. 2023). Using a differentially expressed genes (DEGs) list cutoff, |log2FC= >0 and adjusted *p-*value cutoff of < 0.05, we generated PPI networks for the gene sets for each tissue using the STRING medium confidence required interaction score. When no individual gene was statistically significantly changed after correcting for multiple hypothesis testing, all genes with an uncorrected p-value less than.01 were investigated for over-represented pathways via STRING. These were visualized in STRING with all potential interaction sources used and a moderate level of evidence; enrichment statistics were performed using STRING with the assumption of the whole genome as background. We limited our network generation to only include experimental evidence and evidence from curated databases as active interaction sources. Experimental databases in STRING are (BIND, DIP, GRID, HPRD, IntAct, MINT, and PID), and the curated databases in STRING include the gene ontology (GO) database (Gene Ontology Consortium, 2004), KEGG database (Kanehisa, Goto, 2000), Biocarta (http://www.biocarta.com), BioCyc (Karp et al., 2019) and Reactome (Milacic et al., 2024). Finally, Cytoscape (v 3.9.1) (Shannon et al. 2003) an open-source platform, was used to search whether there were any additional pathways.

## 3. Results

No significant differences were observed in dam water consumption between arsenic-exposed and controls (Supplemental Figure S1). Water consumption was recorded starting at the interval between 10 and 6 days prior to conception. Only at one timepoint (4 days prior to conception: day −4) was there a small but significant difference observed between treatment groups in water consumption; however, the overall trend throughout the experimental period did not indicate a difference in water consumption. Besides the fact that both groups ate less food when they were separated into individual cages at gestational day 8, the overall trend throughout the experimental period did not indicate a difference in food consumption between treatment groups (Supplemental Figure S2). There were no significant differences in weight between the arsenic-exposed dams compared to the controls (Supplemental Figure S3). As there was no difference in water or food intake, or maternal weight gain between treatments in our study, any subsequent findings were not confounded by these variables.

### 3.1. Microarray analysis

**Fig. 2.**
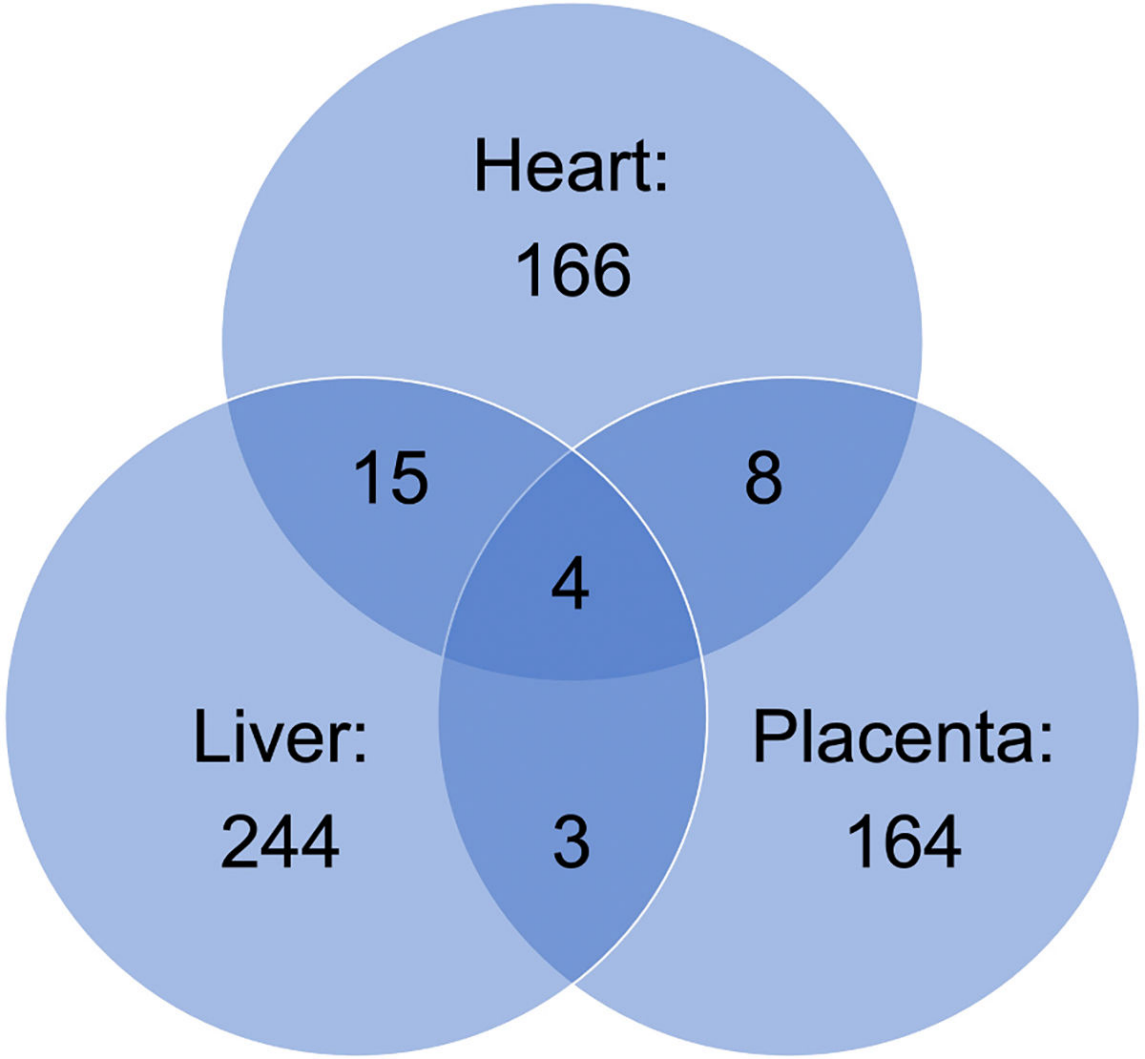
Differential gene expression overlap between heart, liver, and placenta. Tissues including heart, lung, liver, and placenta were excised upon euthanasia of the pregnant dam at gestational day (GD) 18 following preconception and prenatal exposure to either 0 ppb (control) or 100 ppb (exposed) sodium (meta) arsenite. Tissues from the C57Bl/6 J mice were flash frozen using liquid nitrogen and stored at −80˚C. RNA was extracted using kits from Qiagen, reverse transcribed, and gene expression analysis was carried out using the Agilent Whole Mouse 44 K Genome Oligo Array. Among the genes altered with an unadjusted *p*-value < 0.01, only four were found to overlap between placenta, liver, and heart. Similarly, there was minimal overlap between heart and liver (15), liver and placenta (3), and heart and placenta (8). No over-lapping genes were identified between the lung and any other organ. Data shown represents an n = 3.

Raw data from microarray experiments was preprocessed, quantile-normalized and differential gene expression between exposed (100 ppb iAs) and unexposed (0 ppb) was determined for fetal heart (Table S2), liver (Table S3), lung (Table S4) and placenta (Table S5). Of the genes altered with an unadjusted *p*-value < 0.01, just four mapped genes were changed across all tissues: the heart, liver, and placenta (*Pim3*, *Ifit3*, *Psmb8*, and *Rtp4*; Fig. 2). Between the heart and liver, overlap occurred in 15 genes: *C1qb, Tyrobp, Hic1, Ankrd37, Ly6c1, Gm16430, Isg15, Ifitm3, Gcm1, Irf9, Usp18, Oas1a, Oasl1, Xaf1,* and *Gzma*. Heart and placenta had eight of the same differentially expressed genes (*Hmga1, Irgm2, Irgm1, Tmem140, Pde4b, Ifi204, Gbp3,* and *Cmpk2*). Liver and placenta had just three overlapping genes (*Tgtp2, Pfkfb3*, and *Dek*). While 49 genes were found to be differentially expressed in the lung, none over-lapped with any of the other organs studied. After the altered genes for each organ were input into STRING and a map was acquired, dysregulated biological pathways were identified. When querying potential impact of prenatal arsenic exposure on the glucocorticoid receptor pathway, 9 (*Aqp1, H6pd, Cyb561, Zhx3, Slc22a5, Pdgfrb, Sgk1, Spsb1*, and *Aff1*) out of 12 previously identified genes associated with arsenic exposure (Meakin et al. 2019) were dysregulated in fetal liver, heart, lung, and placenta tissues (Supplemental Figure S4 and Supplemental Table S6).

#### 3.1.1. Fetal liver

**Fig. 3.**
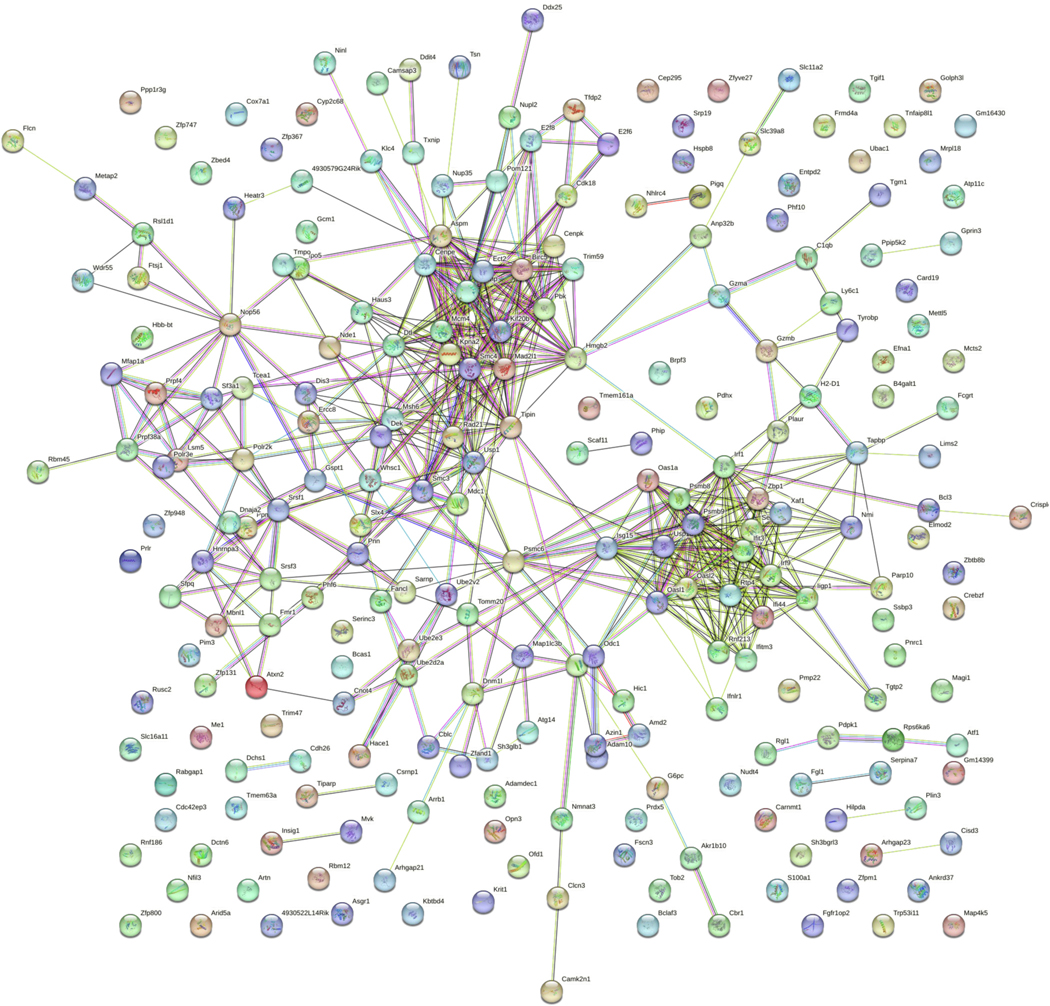
Liver STRING map. Liver samples were obtained from gestational day (GD) 18 C57Bl/6 J fetal mice exposed for 2 weeks prior to conception and during gestation to either 0 ppb (control) or 100 ppb (exposed) sodium (meta) arsenite. After microarray analysis, 240 differentially expressed (comparing exposed to controls exhibited an unadjusted *p*-value <0.01) genes from the liver were entered into the STRING database to generate a map. Nodes are indicated by the colored circles; lines indicate connections between nodes. Data shown represents an n = 3.

Findings from the liver revealed 251 differentially expressed transcripts, of which 240 were known and could be input into STRING (Fig. 3). In the microarray, Interferon-induced transmembrane protein 3 (*Ifitm3*) was downregulated in arsenic-exposed mice (−1.02 log fold change). Interestingly, *Ifitm3* and *Psmb8* were two of the genes found to be highly connected to other altered transcripts. Enriched biological processes included regulation of biological process, cellular process, and biological regulation (top 10 results in Table 2, complete list in Supplemental Table S7). No KEGG pathway enrichment was identified in the STRING database based on the input. After inputting the same differentially expressed transcripts into Cytoscape, the human pathway related to immune response to tuberculosis (WP4197) was identified. In particular, the downstream regulation of this pathway includes *Psmb8*, *Ifit3*, and interferon regulatory factors (*Irf*) 1 & 9, which were found to be differentially regulated in the current mouse model.

#### 3.1.2. Fetal heart

**Fig. 4.**
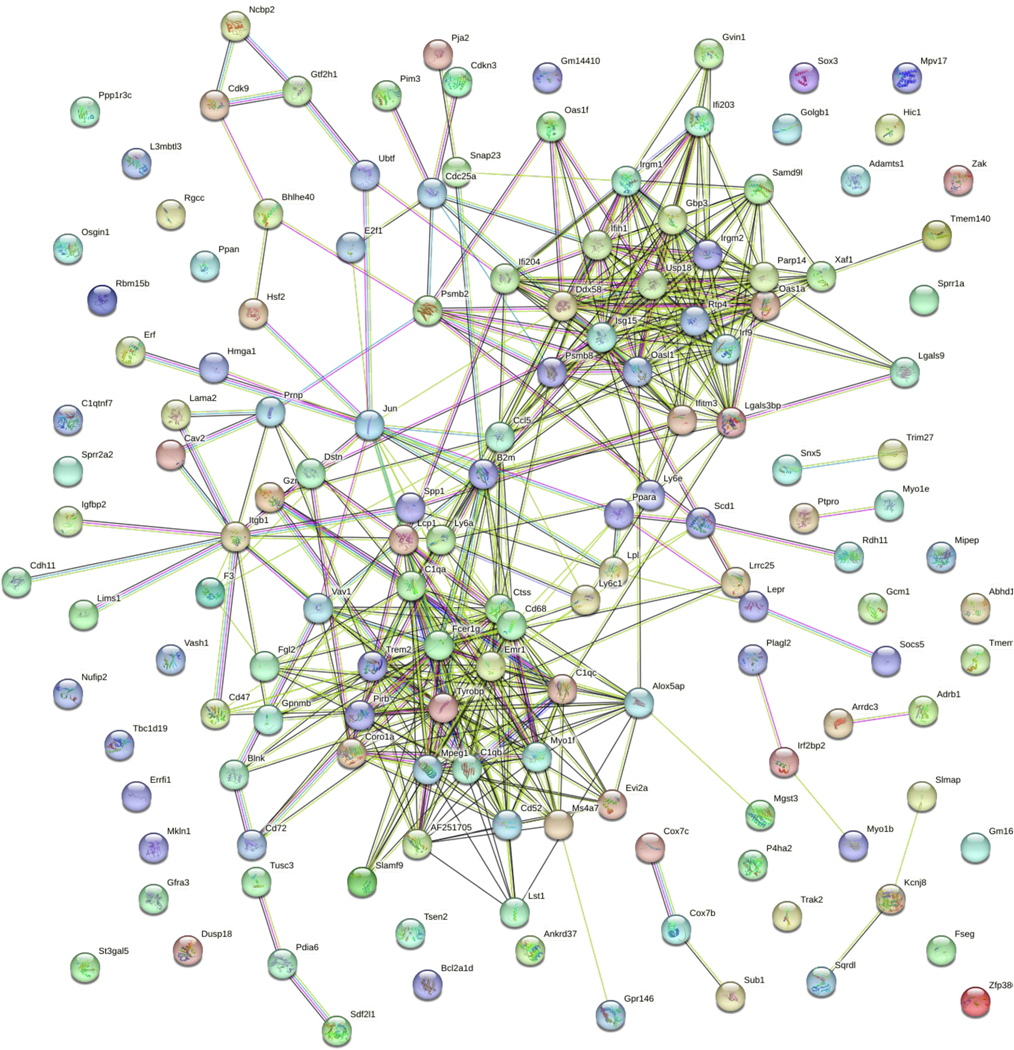
Heart STRING map. Heart samples were obtained from gestational day (GD) 18 C57Bl/6 J fetal mice exposed for 2 weeks prior to conception and during gestation to either 0 ppb (control) or 100 ppb (exposed) sodium (meta) arsenite. After microarray analysis, 156 differentially expressed (comparing exposed to controls exhibited an unadjusted *p*-value <0.01) genes from the heart were entered into the STRING database to generate a map. Nodes are indicated by the colored circles; lines indicate connections between nodes. Data shown represents an n = 3.

Data from the heart revealed 163 differentially expressed RNA transcripts, of which 150 known transcripts were entered into STRING. A STRING interaction map was generated (Fig. 4) and information was obtained on enriched biological processes (based on gene ontology - GO) and KEGG pathways (top 10 STRING/GO findings in Table 3, all KEGG findings in Table 4). The three most enriched biological processes were immune system process, immune response, and immune effector process. Similarly, enriched KEGG pathways revolved mainly around immune response with the top three as prion diseases, osteoclast differentiation, and the B cell receptor signaling pathway. Again, in the heart, *Psmb8* was a highly connected transcript, as was *Ifitm3*. C-C motif chemokine ligand 5 (*Ccl5*) appeared to be a key factor linking various components of the two major networks identified in the STRING diagram. Both *Itgb1* and *Lpl* were slightly outside of the main networks but still connected to many other dysregulated genes. Cytoscape inquiry revealed the human macrophage markers *CD68* and coagulation factor 3 (*F3*) as two of the dysregulated genes related to the WikiPathway WP4146 – Macrophage markers.

#### 3.1.3. Fetal lung

**Fig. 5.**
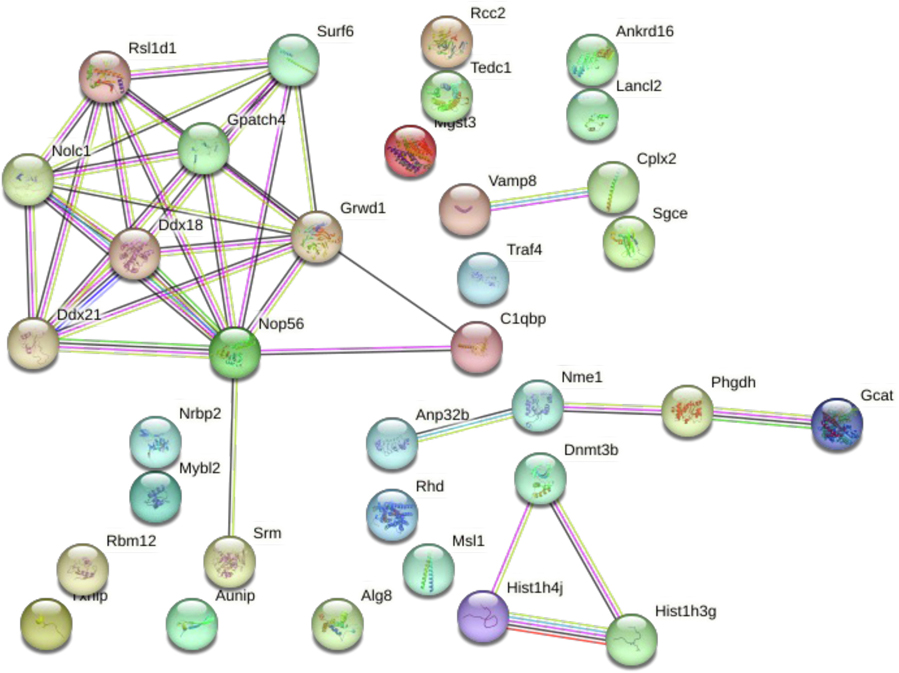
Lung STRING map. Lung samples were obtained from gestational day (GD) 18 C57Bl/6 J fetal mice exposed for 2 weeks prior to conception and during gestation to either 0 ppb (control) or 100 ppb (exposed) sodium (meta) arsenite. After microarray analysis, 41 differentially expressed (comparing exposed to controls exhibited an unadjusted *p*-value <0.01) genes from the lung were entered into the STRING database to generate a map. Nodes are indicated by colored circles; lines denote connections between nodes. Data shown represents an n = 3.

A total of 49 mRNAs were altered in lung tissue, with 41 of these being input into the STRING program. *Nop56* was one of the most connected transcripts identified in the generated STRING map for the lung. Although a small network of genes was identified to be interacting (Fig. 5), four biological processes were found to be enriched within the set including chromatin assembly, ribosome biogenesis, cellular component biogenesis, and nucleosome assembly (Table 5A). Just one KEGG pathway was enriched; cysteine and methionine metabolism (Table 5B). Similarly, analysis in Cytoscape mapped DNA methyltransferase 3B (*Dnmt3b*) and 3-phosphoglycerate dehydrogenase *3.1.4.Placenta*(*Phgdh*) to WP2525, the pathway for trans-sulfuration and one-carbon In the placenta, there were 190 differentially expressed mRNAs; 165 metabolism.of these were input into STRING because many of them were not mapped to a known transcript. There was one more highly connected area in methyltransferase 3B (*Dnmt3b*) and 3-phosphoglycerate dehydrogenase (*Phgdh*) to WP2525, the pathway for trans-sulfuration and one-carbon metabolism.

#### 3.1.4. Placenta

**Fig. 6.**
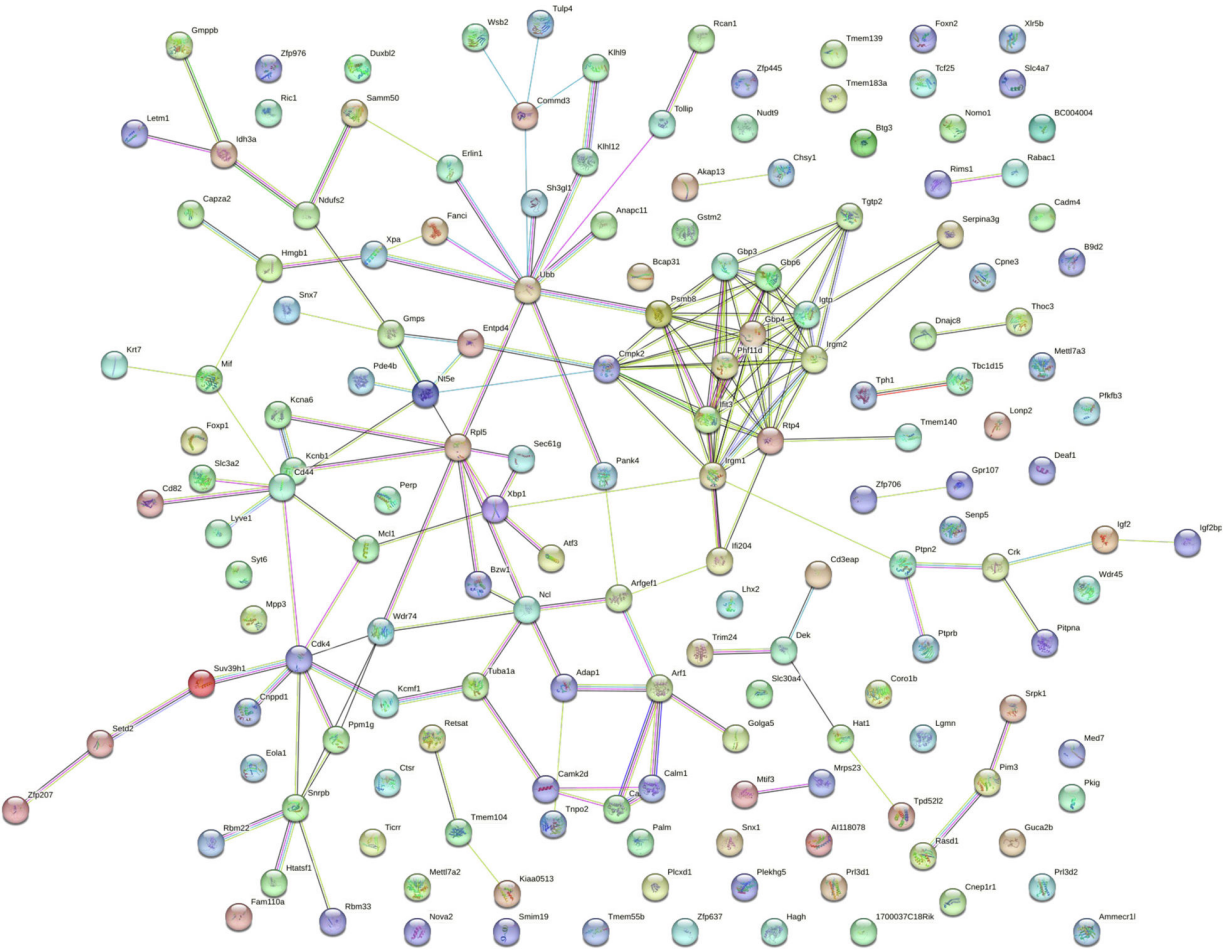
Placenta STRING map. Placenta samples were obtained from gestational day (GD) 18 C57Bl/6 J fetal mice exposed for 2 weeks prior to conception and during gestation to either 0 ppb (control) or 100 ppb (exposed) sodium (meta) arsenite. After microarray analysis, 165 differentially expressed (comparing exposed to controls exhibited an unadjusted *p*-value <0.01) genes from the placenta were entered into the STRING database to generate a map. Nodes are indicated by colored circles; lines denote connections between nodes. Data shown represents an n = 3.

In the placenta, there were 190 differentially expressed mRNAs; 165 of these were input into STRING because many of them were not mapped to a known transcript. There was one more highly connected area in the STRING map and many of those connections included guanylate-binding protein 3 (*Gbp3*) and proteasome subunit beta type-8 (*Psmb8*) (Fig. 6). Although there were no GO or KEGG pathways found to be enriched in this data, enriched cellular components were identified (Table 6). After input into Cytoscape, the same pathway identified for liver tissue exhibited similarity in the dysregulated genes for placenta tissue. Specifically, interferon-induced protein with tetratricopeptide repeats 3 (*Ifit3*), *Psmb8*, and protein tyrosine phosphatase, non-receptor type 2 (*Ptpn2*) mapped to this pathway.

### 3.2. RT-qPCR analysis

**Fig. 7.**
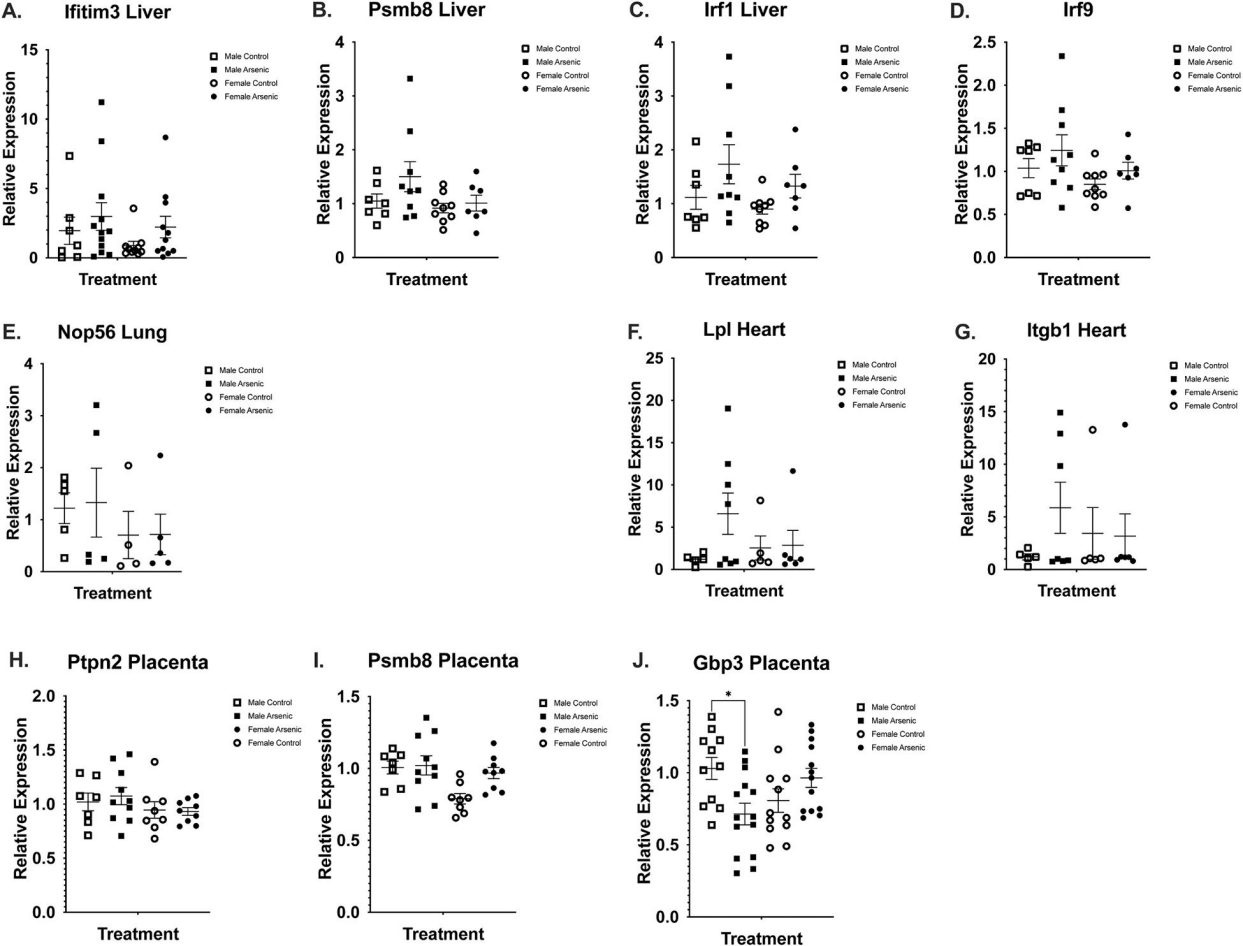
RT-qPCR Analysis of select genes in lung, liver, heart, and placenta of GD18 mice offspring prenatally exposed to arsenic. Tissues including heart, lung, liver, and placenta were excised upon euthanasia of the pregnant dam at gestational day (GD) 18 following preconception and prenatal exposure to either 0 ppb (control) or 100 ppb (exposed) sodium (meta) arsenite. Tissues from the C57Bl/6 J mice were flash frozen using liquid nitrogen and stored at −80˚C. RNA was extracted using Qiagen kits, reverse transcribed, and gene expression analysis was carried out using RT-qPCR. Select genes (*Ifitm3, Psmb8, Irf1, Irf9 in liver, Lpl* and *Itgb1* in heart, *Nop56* in lung, and *Gbp3, Psmb8, Ptpn2* in placenta) were analyzed by RT-qPCR to examine organ-specific alterations in gene expression. All runs were normalized to 18S ribosomal RNA. Data shown represents an n = 2–15 out of 2–6 different litters (Supplemental Table 1). No findings reached statistical significance.

Selected genes based on prior arsenic exposure literature and relevant nodes from the microarray analysis (below) were analyzed using RT-qPCR to examine organ-specific alterations in gene expression following preconception and prenatal arsenic exposure. Although no significance was discovered, there was a trending association in placenta. In the liver, interferon induced transmembrane protein 3 (*Ifitm3*), proteasome subunit beta type-8 (*Psmb8*), interferon regulatory factor 1 (*Irf1*) and interferon regulatory factor 9 (*Irf9*) were unchanged when exposure groups were compared to controls (Fig. 7, A-D). Nop56 ribonucleoprotein (*Nop56*) expression in lung tissue was also unaffected by exposure, with some samples showing higher expression than others, but no major trends were observed across exposure groups (Fig. 7E). In the heart, no significant differences were observed in relative expression of lipoprotein lipase (*Lpl*) or integrin beta 1 (Itgb1) (Fig. 7, F & G). One sample in the male arsenic group had higher expression of 18S ribosomal RNA compared to other samples (still within two standard deviations of the mean) which resulted in much higher 2^ΔΔCt values for both heart mRNAs. In placenta, no significant differences were observed in relative expression of protein tyrosine phosphatase non-receptor type 2 (*Ptpn2)* (Fig. 7H) or *Psmb8* (Fig. 7I). Interestingly, guanylate-binding protein 3 (*Gbp3*) expression in placenta did reach significance (*p* = 0.0237) when comparing male arsenic-exposed placentas to controls (Fig. 7J).

## 4. Discussion

Although *Ifitm3* was not found to be significantly different in the liver of this model upon examination by RT-PCR, it was one of the most differentially expressed transcripts in the microarray and was highly connected in the STRING map. This may indicate that investigation of a single transcript in response to low level arsenic exposure is not sufficient to display a complete portrait of effects. Furthermore, in both the liver and placenta, this dysregulated gene, along with *Psmb8*, mapped to the pathway involved in immune response to tuberculosis. Similarly, *Ifitm3* normally acts to inhibit viral entry into host cell cytoplasm (Li et al. 2013; Narayana et al. 2015); therefore, as a potential mechanism of immune suppression following prenatal arsenic exposure, it is of interest for further investigation. *Ifitm3* was one of just a few genes found to be altered in the heart, liver, and placenta and has been identified as an intrinsic factor which can be upregulated by interferons (Jiang et al. 2018). It is also recognized as a limiting factor in influenza pathogenesis (Everitt et al. 2012) and seems to play a role in human metastasis prevention (Gómez-Herranz et al. 2019). Found to be downregulated in chronically iAs-treated hamsters (Hernández et al. 2011), Ifitm3 was identified as a potential mediator of hepatic carcinogenesis. Despite the fact that the exposure level was significantly higher in the hamsters (15 mg/L or 15,000 ppb), *Ifitm3* and related pathways represent potential targets for mechanistic intervention. Furthermore, given the data on long-term susceptibility to tuberculosis following prenatal exposure to arsenic, *Ifitm3* and *Psmb8* may be mediators, along with other intrinsic factors, of those long-term effects.

*Psmb8*, or proteasome 20S subunit beta 8, is a key component of MHC class II and is expressed in many tissues in the body (PubChem gene 16913) (PubChem, 2024). It is induced by interferon gamma and, interestingly, mutation in this subunit of the immunoproteasome has been linked to increased inflammation and lipodystrophy (Kitamura et al. 2011). Results from the current inquiry reveal a small downregulation of *Psmb8* in the placenta, heart, and liver. Prior work found no effect of arsenic trioxide on expression of *Psmb8* in NB4 acute promyelocytic leukemia cells; however, in the same study, a reduction in promoter activity of *Psmb8* was noted (Yang et al. 2014).

Despite setting out to discover the relationship between prenatal arsenic exposure and the glucocorticoid receptor signaling pathway, the results highlight the known link between arsenic and impaired lipid metabolism (Renu et al. 2018), which is, of course, also linked to macrophage function. *Nop56*, found to be dysregulated in the lung of this model, has previously been found to be associated with metal exposure (including arsenic) and atherosclerosis (Riffo-Campos et al. 2018). Prenatal exposure has previously been linked to altered signaling in the liver, contributing to earlier onset of atherosclerosis in the apolipoprotein E-knockout mouse model (States et al. 2012). *Lpl* encodes a water-soluble enzyme important in lipoprotein processing, an essential macrophage function. It was not found to be different after RT-PCR analysis but was identified as an altered transcript in the microarray. Interestingly, *Lpl* was found to be down-regulated following arsenic exposure due to its suppressive effect (arguably, toxicity) to macrophages (Song et al. 2019). Interestingly, recent work has shown that a drug inhibiting lipase activity can reverse these effects (Lou et al. 2022).

Other findings were less specific, but many still are related to the immune response. For instance, *Itgb1*, involved in cell adhesion and recognition for multiple processes including immune response, did not show significant differences in RT-PCR analysis but exhibited altered expression in the microarray results. Further, this member of the arrestin family of proteins was found to be positively associated with arsenic exposure in two Bangladeshi cohorts (Demanelis et al. 2019). *Gbp3* (−0.43 log FC), a transcript previously found to be upregulated in arsenic-exposed fetal C3H mouse liver (Liu et al. 2007), is one of a group of GTPases upregulated in response to interferons (Tretina et al., 2019). Evidence indicates that this variant acts to moderate influenza virus, decreasing viral activity within the cell (Nordmann et al. 2012). In this study, a decrease in *Gbp3* was observed in the placenta and liver, in alignment with findings of larger immune pathway downregulation and increased susceptibility to respiratory infection observed in human populations following prenatal arsenic exposure (Farzan et al. 2016; Laine and Fry, 2016).

While not explicitly isolated as a pathway in any analysis of the microarray, 9 of the 12 genes found to be altered within the glucocorticoid receptor pathway in JEG-3 choriocarcinoma cells by Meakin et al., 2019 were also dysregulated in the microarray results from the current study’s liver, heart, lung, and placenta tissues. Namely, aquaporin 1 (*Aqp1*), Hexose-6-dehydrogenase (*H6pd*), Cytochrome b-561 (*Cyb561*), zinc fingers and homeoboxes protein 3 (*Zhx3*), solute carrier family 22 (organic cation/carnitine transporter) member 5 (*Slc22a5*), platelet-derived growth factor receptor beta (*Pdgfrb*), serum/glucocorticoid-regulated kinase 1 (*Sgk1*), splA/ryanodine receptor domain and SOCS box containing 1 (*Spsb1*), and AF4/FMR2 family, member 1 (*Aff1*) were all dysregulated in the microarray data, confirming prior findings in a relevant cell model but extending those findings to an organism-level exposure method and four organs at gestational day 18.

Importantly, these findings underline the idea that although small changes may be observed in individual genes across organs, these minute alterations across a network can lead to larger ramifications at the system level. This idea of network dynamics is influencing the field of medicine and allowing for use of artificial intelligence in order to make predictions about disease and exposure based on transcriptomic data (Theodoris et al. 2023). Using the presented data, a comparison across organs, in conjunction with other studies allows for correlation and furthers understanding of the mechanistic basis for long-term effects of preconception and prenatal arsenic exposure.

While mouse models are informative, they may not fully reflect human responses to arsenic exposure. However, mouse models are a key tool to investigate mechanisms of effects and provide insight into potential routes of intervention that could reduce long-term effects following early life exposure. The C57Bl/6 model has previously been used to characterize long-term effects of prenatal arsenic exposure and revealed various effects on the lungs and immune system (Rychlik et al. 2023), two systems well known to be affected by arsenic exposure. The current study set out to examine gene transcription at gestational day 18, which may not capture dynamic gene expression changes throughout development; however, in order to examine effects in multiple organs for comparison, this time point is both practical and of use to link gene expression alterations in the placenta to those in other organs associated with long-term phenotypes that have been previously characterized. Although protein was not analyzed in the current study, mRNA and protein levels are very often substantially correlated (Buccitelli and Selbach, 2020), and when viewed as an intermediary and potential target for intervention, mRNA is a highly relevant and useful marker for gene expression and as a correlate to protein function. Finally, the scope of this research included a single arsenic dose. These limitations prevent assessment of a dose-response relationship; however, as discussed in the introduction, the dose selected is relevant to many human populations (Naujokas et al. 2013; Chen and Costa, 2021; García Salcedo et al. 2022).

**Fig. 8.**
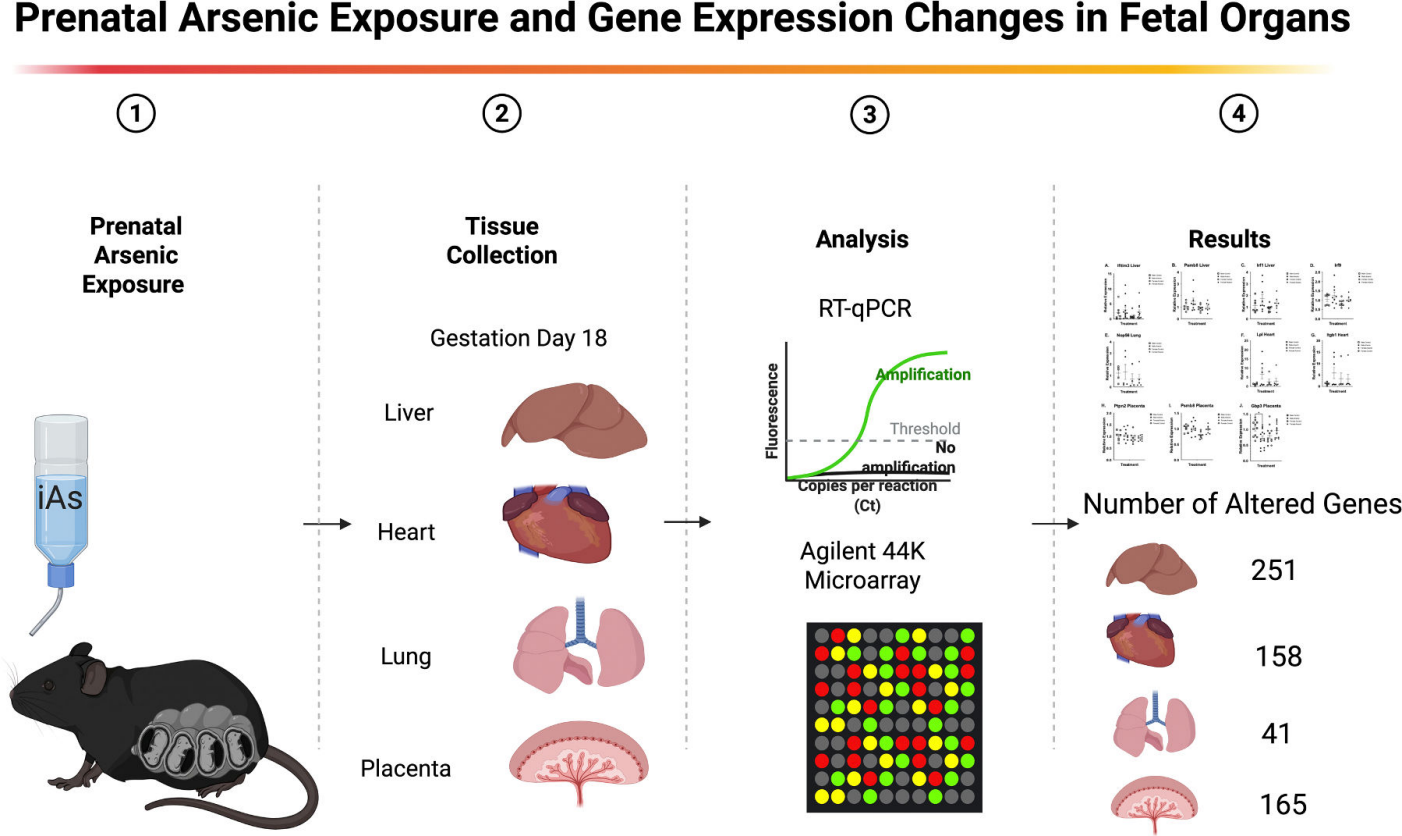
Prenatal arsenic exposure and gene expression changes in fetal organs. Graphical abstract: Female C57BL/6 J mice were exposed to inorganic arsenic (iAs) in drinking water during pregnancy, followed by tissue collection at gestation day (GD) 18 from fetal liver, heart, lung, and placenta. Gene expression was assessed using RT-qPCR and untargeted Agilent 44 K microarray profiling, with downstream bioinformatics and network/pathway enrichment analyses (STRING queried and visualized in Cytoscape). Prenatal arsenic exposure resulted in differential mRNA expression across all organs, with 251 altered genes in liver, 165 in placenta, 158 in heart, and 41 in lung compared with controls. Altered pathways were predominantly immune-related, and placentas from male fetuses showed reduced expression of guanylate-binding protein 3 (*Gbp3*) expression in placenta (p = 0.0237) when comparing male arsenic-exposed placentas to controls.

To our knowledge this is the first publication making a comparison of gene expression alterations in multiple organs following prenatal exposure to a relevant level of arsenic. Prenatal arsenic exposure at an environmentally relevant concentration produced measurable, organ-specific shifts in fetal gene expression at GD18, with the liver, placenta, and heart showing the largest number of altered transcripts and the lung showing a more limited response (See graphical abstract in Fig. 8). Across tissues, pathway enrichment consistently pointed to immune-related signaling, reinforcing the idea that immune programming is a key early target of in utero arsenic exposure and a plausible mechanistic link to later-life immune and inflammatory outcomes reported in exposed populations. Notably, the placenta emerged as a potential hub for sex-dependent effects, with reduced placental expression of Gbp3 in male fetuses suggesting altered interferon gamma–related responsiveness at the maternal–fetal interface. The reduced *Gbp3* in male fetuses likely results from a complex interplay between arsenic’s epigenetic effects, which are known to be sex-specific (Niedzwiecki et al. 2015; Caldwell et al. 2018; Winterbottom, Moroishi, et al. 2019), and through genetic and/or hormonal contributions to the sexual dimorphism of fetal and placental immunity (Baines and West, 2023). Together, these data demonstrate that prenatal arsenic exposure per-turbs immune-relevant transcriptional networks across organs during late gestation, providing a mechanistic foundation for the long-term health effects associated with early-life arsenic exposure while also highlighting the placenta and fetal sex as important variables for follow-up work. Further investigation into dose-response separated by sex and at additional windows of exposure can continue the elucidation of these pathways as potential mechanistic targets to prevent long-term effects of arsenic exposure is needed.

## Supplementary Material

MMC5

MMC6

MMC8

MMC1

MMC2

MMC3

MMC7

MMC9

MMC4

## Figures and Tables

**Table 1:** Primer sets for RT-qPCR.

Gene	Forward	Reverse	RT-PCR Protocol

Ifitm3	CTGAACATCAGCACCTTGGT	TTTTGGTGGTTATCAAGTGCACT	50^◦^C for 30 min 95^◦^C for 2 min 45 cycles: 95^◦^C for 15 s, 61.5^◦^C for 40 s
Lpl	GTGGCCGAGAGCGAGAACAT	GCTTTCACTCGCATCCTCTC	1st cycle: 94^◦^C for 40 s, 55^◦^C for 45 s, 72^◦^C for 1min20s 30 cycles: 94^◦^C for 15 s, 55^◦^C for 45 s, 72^◦^C for 1min20s
Itgb1	TGTGACCCATTGCAAGGAGAAGGA	AATTGGGATGATGTCGGGACCAGT	1st cycle: 94^◦^C for 40 s, 55^◦^C for 45 s, 72^◦^C for 1min20s 30 cycles: 94^◦^C for 15 s, 55^◦^C for 45 s, 72^◦^C for 1min20s
Nop56	GTTGGCGCTGAAGGAAGTGG	CTTTGGCACGAGAGTAGCTG	95^◦^C 2 min 40 cycles: 95^◦^C for 5 s, 55^◦^C for 30 s
Gbp3	TGGAGGCACCCATTTGTCTGGTG	GACAAAGGTGCTGCTCAGAAGCACAG	45 cycles: 95^◦^C for 1 min, 56^◦^C for 1min30s, 72^◦^C for 1min30s
18S Ribosomal	GGGAGCCTGAGAAACGGC	GGGTCGGGAGTGGGTAATTT	Run with each gene primer set protocol

The primer sets listed in the table were utilized for analysis of mRNA for individual genes selected from the most dysregulated genes from the microarray data that demonstrated the most connections in generated STRING maps. All mRNA quantification was normalized to 18S ribosomal mRNA expression.

**Table 2:** Top 10 enriched biological processes in arsenic-exposed GD18 liver tissue.

#Term ID	Term description	Obs gene count	Bkgd gene count	Strength	False discovery rate	Matching proteins in your network (labels)

GO:0050789	Regulation of biological process	163	9973	0.18	1.95E-08	Plaur,Tmem161a,Fancl,Clcn3,Brpf3,Msh6,Gzmb,Serinc3,Pmp22,Kpna2,
						G6pc,Plin3,Tmpo,Ddit4,Ppm1d,E2f6,Dek,Cenpk,Psmc6,Pbk,Rad21,Atf1,
						Slc11a2,Gzma,Gcm1,Phf10,Tapbp,Psmb8,Lims2,Incenp,Prdx5,Smc3,
						Ifitm3,Cdk18,Rgl1,Opn3,Dtl,Entpd2,Nup35,Ube2e3,Zbp1,Efna1,
						B4galt1,Usp1,Ssbp3,Sfpq,Oasl1,Oasl2,Usp18,Iigp1,Tyrobp,Ipo5,
						Serpina7,Fgl1,Map1lc3b,Ddx25,Phip,Tipin,Me1,Csrnp1,Mvk,Hspb8,
						Zfand1,Slx4,Atg14,Hace1,Serpine1,Cblc,C1qb,Ofd1,Pim3,Card19,
						Tiparp,Rbm12,Zfp367,Elmod2,Hic1,Atxn2,E2f8,Cenpe,Zfpm1,S100a1,
						Whsc1,Aspm,Ercc8,Camk2n1,Hilpda,Tob2,Insig1,Adam10,Artn,Nfil3,
						Azin1,Hmgb2,Ly6c1,Cdc42ep3,Ifnlr1,Frmd4a,Tnfaip8l1,Dchs1,Phf6,
						Gspt1,Krit1,Birc5,Oas1a,Slc39a8,Rps6ka6,Isg15,Kif20b,Fmr1,Magi1,
						Rnf213,Hbb-bt,Arrb1,Cox7a1,Mbnl1,Mad2l1,Atp11c,Flcn,Ifit3,Anp32b,
						Pdpk1,Sirt1,Zbtb8b,Txnip,Crebzf,Trim59,Ect2,Irf1,Map4k5,Hnrnpa3,
						Bclaf3,Cnot4,Arid5a,Dnm1l,Ube2v2,Rnf186,Arhgap23,Rsl1d1,Bcl3,
						Srsf3,Irf9,Srsf1,Nmi,Prlr,Ppp1r3g,Xaf1,Cep295,Camsap3,Lsm5,Tgm1,
						Tfdp2,Odc1,Tcea1,Parp10,Sh3glb1,Zfyve27,Tgif1,Psmb9,Arhgap21,H2-
						D1,Golph3l,Zfp131
GO:0009987	Cellular process	195	13330	0.13	2.93E-08	Sf3a1,Plaur,Tmem161a,Fancl,Clcn3,Brpf3,Msh6,Pdhx,Bcas1,Gzmb,
						Serinc3,Pmp22,Kpna2,G6pc,Nudt4,Ddit4,Ppm1d,E2f6,Trim47,Pnn,Dek,
						Cenpk,Psmc6,Pbk,Rad21,Mcm4,Nde1,Atf1,Slc11a2,Gzma,Gcm1,Phf10,
						Tapbp,Psmb8,Lims2,Incenp,Carnmt1,Prdx5,Smc3,Ifitm3,Pigq,Tsn,
						Cdk18,Rgl1,Tmem63a,Opn3,Dtl,Entpd2,Nup35,Ube2e3,Zbp1,Efna1,
						B4galt1,Usp1,Ssbp3,Sfpq,Sh3bgrl3,Oasl2,Fscn3,Usp18,Iigp1,Tyrobp,
						Ipo5,Polr3e,Dctn6,Fgl1,Heatr3,Dnaja2,Map1lc3b,Ddx25,Phip,Tipin,
						Me1,Csrnp1,Mvk,Hspb8,Zfand1,Slx4,Wdr55,Atg14,Hace1,Serpine1,
						Cblc,Ubac1,C1qb,Rbm45,Rtp4,Ofd1,Dis3,Pim3,Smc4,Tiparp,Cdh26,
						Cbr1,Haus3,Mettl5,Polr2k,Hic1,Atxn2,E2f8,Cenpe,Zfpm1,Whsc1,Aspm,
						Ercc8,Hilpda,Insig1,Nupl2,Adam10,Artn,Nfil3,Azin1,Hmgb2,Ly6c1,
						Cdc42ep3,Rabgap1,Srp19,Ifnlr1,Frmd4a,Dchs1,Mrpl18,Prpf38a,Gspt1,
						Krit1,Birc5,Oas1a,Amd2,Slc39a8,Rps6ka6,Mdc1,Prpf4,Nhlrc4,Isg15,
						Kif20b,Fmr1,Magi1,Mfap1a,Asgr1,Rnf213,Hbb-bt,Arrb1,Cox7a1,Mbnl1,
						Mad2l1,Atp11c,Nop56,Flcn,Ifit3,Anp32b,Pdpk1,Mcts2,Sirt1,Txnip,
						Crebzf,Trim59,Ect2,Irf1,Ninl,Map4k5,Pom121,Hnrnpa3,Ppip5k2,
						Nmnat3,Cnot4,Arid5a,Dnm1l,Ube2v2,Rnf186,Arhgap23,Rsl1d1,Bcl3,
						Slc16a11,Srsf3,Srsf1,Nmi,Prlr,Crispld2,Xaf1,Camsap3,Lsm5,Tgm1,
						Tfdp2,Odc1,Tcea1,Parp10,Sh3glb1,Zfyve27,Tgif1,Ube2d2a,Psmb9,
						Arhgap21,H2-D1,Golph3l,Tomm20,Metap2
GO:0065007	Biological regulation	167	10591	0.16	6.45E-08	Plaur,Tmem161a,Fancl,Clcn3,Brpf3,Msh6,Gzmb,Serinc3,Pmp22,Kpna2,
						G6pc,Plin3,Tmpo,Ddit4,Ppm1d,E2f6,Dek,Cenpk,Psmc6,Pbk,Rad21,
						Nde1,Atf1,Slc11a2,Gzma,Gcm1,Phf10,Tapbp,Psmb8,Lims2,Incenp,
						Prdx5,Smc3,Ifitm3,Cdk18,Rgl1,Opn3,Dtl,Entpd2,Nup35,Ube2e3,Zbp1,
						Efna1,B4galt1,Usp1,Ssbp3,Sfpq,Oasl1,Oasl2,Usp18,Iigp1,Tyrobp,Ipo5,
						Serpina7,Fgl1,Dnaja2,Map1lc3b,Ddx25,Phip,Tipin,Me1,Csrnp1,Mvk,
						Hspb8,Zfand1,Slx4,Atg14,Hace1,Serpine1,Cblc,C1qb,Ofd1,Dis3,Pim3,
						Card19,Tiparp,Rbm12,Zfp367,Elmod2,Hic1,Atxn2,E2f8,Cenpe,Zfpm1,
						S100a1,Whsc1,Aspm,Ercc8,Camk2n1,Hilpda,Tob2,Insig1,Adam10,Artn,
						Nfil3,Azin1,Hmgb2,Ly6c1,Cdc42ep3,Rabgap1,Ifnlr1,Frmd4a,Tnfaip8l1,
						Dchs1,Phf6,Gspt1,Krit1,Birc5,Oas1a,Slc39a8,Rps6ka6,Isg15,Kif20b,
						Fmr1,Magi1,Rnf213,Hbb-bt,Arrb1,Cox7a1,Mbnl1,Mad2l1,Atp11c,Flcn,
						Ifit3,Anp32b,Pdpk1,Sirt1,Zbtb8b,Txnip,Crebzf,Trim59,Ect2,Irf1,
						Map4k5,Hnrnpa3,Bclaf3,Cnot4,Arid5a,Dnm1l,Ube2v2,Rnf186,
						Arhgap23,Rsl1d1,Bcl3,Srsf3,Irf9,Srsf1,Nmi,Prlr,Ppp1r3g,Xaf1,Cep295,
						Camsap3,Lsm5,Tgm1,Tfdp2,Odc1,Tcea1,Parp10,Sh3glb1,Zfyve27,Tgif1,
						Psmb9,Arhgap21,H2-D1,Golph3l,Zfp131
GO:0019222	Regulation of metabolic	115	6085	0.24	7.81E-08	Plaur,Tmem161a,Clcn3,Brpf3,Msh6,Pmp22,G6pc,Tmpo,Ddit4,Ppm1d,
	process					E2f6,Dek,Cenpk,Psmc6,Pbk,Rad21,Atf1,Slc11a2,Gzma,Gcm1,Phf10,
						Tapbp,Psmb8,Incenp,Prdx5,Smc3,Cdk18,Dtl,Nup35,Efna1,Usp1,Ssbp3,
						Sfpq,Oasl1,Oasl2,Iigp1,Tyrobp,Serpina7,Fgl1,Ddx25,Phip,Tipin,Me1,
						Csrnp1,Mvk,Hspb8,Slx4,Atg14,Hace1,Serpine1,Cblc,Tiparp,Rbm12,
						Zfp367,Hic1,Atxn2,E2f8,Cenpe,Zfpm1,S100a1,Whsc1,Ercc8,Camk2n1,
						Hilpda,Tob2,Insig1,Nfil3,Azin1,Hmgb2,Phf6,Gspt1,Birc5,Oas1a,Slc39a8,
						Rps6ka6,Isg15,Fmr1,Hbb-bt,Arrb1,Cox7a1,Mbnl1,Mad2l1,Flcn,Anp32b,
						Pdpk1,Sirt1,Zbtb8b,Txnip,Crebzf,Ect2,Irf1,Map4k5,Hnrnpa3,Bclaf3,
						Arid5a,Dnm1l,Ube2v2,Bcl3,Srsf3,Irf9,Srsf1,Nmi,Prlr,Ppp1r3g,Cep295,
						Camsap3,Lsm5,Tfdp2,Odc1,Tcea1,Parp10,Sh3glb1,Tgif1,Psmb9,Zfp131
GO:0060255	Regulation of macromolecule metabolic process	107	5618	0.24	3.81E-07	Plaur,Tmem161a,Brpf3,Msh6,Pmp22,G6pc,Tmpo,Ddit4,Ppm1d,E2f6,
						Dek,Cenpk,Psmc6,Pbk,Rad21,Atf1,Slc11a2,Gzma,Gcm1,Phf10,Tapbp,
						Psmb8,Incenp,Prdx5,Smc3,Cdk18,Dtl,Nup35,Efna1,Usp1,Ssbp3,Sfpq,
						Oasl1,Oasl2,Tyrobp,Serpina7,Ddx25,Phip,Tipin,Csrnp1,Mvk,Slx4,Atg14,
						Hace1,Serpine1,Cblc,Tiparp,Rbm12,Zfp367,Hic1,Atxn2,E2f8,Cenpe,
						Zfpm1,S100a1,Whsc1,Ercc8,Camk2n1,Hilpda,Tob2,Insig1,Nfil3,Azin1,
						Hmgb2,Phf6,Gspt1,Birc5,Oas1a,Slc39a8,Rps6ka6,Isg15,Fmr1,Hbb-bt,
						Arrb1,Mbnl1,Mad2l1,Flcn,Anp32b,Pdpk1,Sirt1,Zbtb8b,Txnip,Crebzf,
						Ect2,Irf1,Map4k5,Hnrnpa3,Bclaf3,Arid5a,Dnm1l,Ube2v2,Bcl3,Srsf3,Irf9,
						Srsf1,Nmi,Prlr,Ppp1r3g,Cep295,Lsm5,Tfdp2,Odc1,Tcea1,Parp10,Tgif1,
						Psmb9,Zfp131
GO:0031323	Regulation of cellular metabolic process	103	5479	0.24	1.94E-06	Plaur,Tmem161a,Clcn3,Brpf3,Msh6,Pmp22,Tmpo,Ddit4,Ppm1d,E2f6,
						Dek,Cenpk,Psmc6,Pbk,Rad21,Atf1,Slc11a2,Gzma,Gcm1,Phf10,Incenp,
						Prdx5,Smc3,Cdk18,Nup35,Efna1,Usp1,Ssbp3,Sfpq,Oasl1,Oasl2,Iigp1,
						Tyrobp,Serpina7,Fgl1,Ddx25,Phip,Tipin,Csrnp1,Mvk,Hspb8,Slx4,Atg14,
						Serpine1,Cblc,Rbm12,Zfp367,Hic1,Atxn2,E2f8,Cenpe,Zfpm1,S100a1,
						Whsc1,Ercc8,Camk2n1,Tob2,Insig1,Nfil3,Hmgb2,Phf6,Gspt1,Birc5,
						Oas1a,Slc39a8,Rps6ka6,Isg15,Fmr1,Hbb-bt,Arrb1,Cox7a1,Mbnl1,
						Mad2l1,Flcn,Anp32b,Pdpk1,Sirt1,Zbtb8b,Txnip,Crebzf,Ect2,Irf1,
						Map4k5,Hnrnpa3,Bclaf3,Arid5a,Dnm1l,Ube2v2,Bcl3,Srsf3,Irf9,Srsf1,
						Nmi,Prlr,Ppp1r3g,Cep295,Camsap3,Tfdp2,Tcea1,Parp10,Sh3glb1,Tgif1,Zfp131
GO:0050794	Regulation of cellular process	151	9541	0.16	1.94E-06	Plaur,Tmem161a,Fancl,Clcn3,Brpf3,Msh6,Gzmb,Serinc3,Pmp22,Kpna2,
						Tmpo,Ddit4,Ppm1d,E2f6,Dek,Cenpk,Psmc6,Pbk,Rad21,Atf1,Slc11a2,
						Gzma,Gcm1,Phf10,Lims2,Incenp,Prdx5,Smc3,Ifitm3,Cdk18,Rgl1,Opn3,
						Dtl,Entpd2,Nup35,Zbp1,Efna1,B4galt1,Usp1,Ssbp3,Sfpq,Oasl1,Oasl2,
						Iigp1,Tyrobp,Ipo5,Serpina7,Fgl1,Ddx25,Phip,Tipin,Csrnp1,Mvk,Hspb8,
						Zfand1,Slx4,Atg14,Hace1,Serpine1,Cblc,Ofd1,Pim3,Card19,Tiparp,
						Rbm12,Zfp367,Hic1,Atxn2,E2f8,Cenpe,Zfpm1,S100a1,Whsc1,Aspm,
						Ercc8,Camk2n1,Hilpda,Tob2,Insig1,Adam10,Artn,Nfil3,Azin1,Hmgb2,
						Ly6c1,Cdc42ep3,Ifnlr1,Frmd4a,Tnfaip8l1,Dchs1,Phf6,Gspt1,Krit1,Birc5,
						Oas1a,Slc39a8,Rps6ka6,Isg15,Kif20b,Fmr1,Magi1,Rnf213,Hbb-bt,Arrb1,
						Cox7a1,Mbnl1,Mad2l1,Atp11c,Flcn,Ifit3,Anp32b,Pdpk1,Sirt1,Zbtb8b,
						Txnip,Crebzf,Trim59,Ect2,Irf1,Map4k5,Hnrnpa3,Bclaf3,Cnot4,Arid5a,
						Dnm1l,Ube2v2,Rnf186,Arhgap23,Rsl1d1,Bcl3,Srsf3,Irf9,Srsf1,Nmi,Prlr,
						Ppp1r3g,Xaf1,Cep295,Camsap3,Tgm1,Tfdp2,Odc1,Tcea1,Parp10,
						Sh3glb1,Zfyve27,Tgif1,Arhgap21,H2-D1,Golph3l,Zfp131
GO:0080090	Regulation of primary metabolic process	100	5290	0.24	2.46E-06	Plaur,Tmem161a,Msh6,Pmp22,Tmpo,Ddit4,Ppm1d,E2f6,Dek,Cenpk,
						Psmc6,Pbk,Rad21,Atf1,Slc11a2,Gzma,Gcm1,Phf10,Psmb8,Incenp,Prdx5,
						Cdk18,Dtl,Nup35,Efna1,Usp1,Ssbp3,Sfpq,Oasl1,Oasl2,Serpina7,Fgl1,
						Ddx25,Phip,Csrnp1,Mvk,Slx4,Atg14,Hace1,Serpine1,Cblc,Tiparp,
						Rbm12,Zfp367,Hic1,E2f8,Cenpe,Zfpm1,S100a1,Whsc1,Ercc8,Camk2n1,
						Tob2,Insig1,Nfil3,Azin1,Hmgb2,Phf6,Gspt1,Birc5,Oas1a,Slc39a8,
						Rps6ka6,Isg15,Fmr1,Hbb-bt,Arrb1,Mbnl1,Mad2l1,Flcn,Anp32b,Pdpk1,
						Sirt1,Zbtb8b,Txnip,Crebzf,Ect2,Irf1,Map4k5,Hnrnpa3,Bclaf3,Arid5a,
						Dnm1l,Ube2v2,Bcl3,Srsf3,Irf9,Srsf1,Nmi,Prlr,Ppp1r3g,Cep295,Tfdp2,
						Odc1,Tcea1,Parp10,Sh3glb1,Tgif1,Psmb9,Zfp131
GO:0009615	Response to virus	17	235	0.82	3.28E-06	Serinc3,Ddit4,Ifitm3,Zbp1,Oasl1,Oasl2,Polr3e,Rtp4,Ifnlr1,Oas1a,Isg15,
						Fmr1,Ifit3,Crebzf,Irf1,Bcl3,Odc1
GO:0051171	Regulation of nitrogen compound metabolic process	97	5126	0.24	4.02E-06	Plaur,Tmem161a,Msh6,Pmp22,Tmpo,Ddit4,Ppm1d,E2f6,Dek,Cenpk,
						Psmc6,Pbk,Rad21,Atf1,Slc11a2,Gzma,Gcm1,Phf10,Psmb8,Incenp,Prdx5,
						Cdk18,Dtl,Nup35,Efna1,Usp1,Ssbp3,Sfpq,Oasl1,Oasl2,Serpina7,Ddx25,
						Phip,Csrnp1,Mvk,Slx4,Atg14,Hace1,Serpine1,Cblc,Tiparp,Rbm12,
						Zfp367,Hic1,E2f8,Cenpe,Zfpm1,S100a1,Whsc1,Ercc8,Camk2n1,Tob2,
						Insig1,Nfil3,Azin1,Hmgb2,Phf6,Gspt1,Birc5,Oas1a,Slc39a8,Rps6ka6,
						Isg15,Fmr1,Hbb-bt,Arrb1,Mbnl1,Mad2l1,Flcn,Anp32b,Pdpk1,Sirt1,
						Zbtb8b,Txnip,Crebzf,Ect2,Irf1,Map4k5,Hnrnpa3,Bclaf3,Arid5a,Dnm1l,
						Ube2v2,Bcl3,Srsf3,Irf9,Srsf1,Nmi,Prlr,Cep295,Tfdp2,Odc1,Tcea1,
						Parp10,Tgif1,Psmb9,Zfp131

Liver samples were obtained from gestational day (GD) 18 C57Bl/6 J fetal mice exposed for 2 weeks prior to conception and during gestation to either 0 ppb (control) or 100 ppb (exposed) sodium (meta) arsenite. After microarray analysis, enriched biological processes were identified based on the 240 differentially expressed (comparing exposed to controls exhibited an unadjusted *p*-value <0.01) genes from the liver using gene ontology (GO) in the STRING database. The specific GO number is reported along with its term description. Each protein from the differentially expressed input that are associated with that biological process are listed in the right column of the table. Data shown represents an n = 3.

**Table 3:** Top 10 enriched biological processes in arsenic-exposed GD18 heart tissue.

#Term ID	Term description	Obs gene count	Bkgd gene count	Strength	False discovery rate	Matching proteins in your network (labels)

GO:0002376	immune system process	45	1703	0.58	7.58E-12	Ebi3,Vav1,Ctss,Trim27,Rgcc,Trem2,Psmb8,Ifitm3,Ifih1,Oasl1,Kcnj8,Tyrobp,Coro1a,Myo1e,C1qc,Lepr,L3mbtl3,Parp14,Ccl5,C1qb,
						Rtp4,Ddx58,C1qa,Irgm1,Blnk,Ptpro,Pirb,Fcer1g,Oas1a,Isg15,Emr1,Myo1f,Itgb1,AF251705,Lst1,B2m,Cd47,Gbp3,Jun,Lgals9,Spp1,
						Snap23,Samd9l,Lcp1,Pja2
GO:0006955	immune response	30	914	0.68	2.33E-09	Vav1,Ctss,Trim27,Rgcc,Trem2,Ifitm3,Ifih1,Oasl1,Tyrobp,Coro1a,C1qc,Parp14,Ccl5,C1qb,Ddx58,C1qa,Irgm1,Blnk,Pirb,Fcer1g,
						Oas1a,Isg15,Emr1,Myo1f,Lst1,B2m,Gbp3,Snap23,Lcp1,Pja2
GO:0002252	immune effector process	20	395	0.87	8.36E-09	Rgcc,Ifitm3,Ifih1,Oasl1,Kcnj8,Tyrobp,Coro1a,C1qc,C1qb,Rtp4,Ddx58,C1qa,Pirb,Fcer1g,Oas1a,Isg15,Myo1f,Cd47,Snap23,Lcp1
GO:0050896	response to stimulus	86	6616	0.28	8.36E-09	Cav2,Ebi3,Ppan,Vav1,Gtf2h1,Cdk9,Ctss,Lpl,Lims1,Vash1,Trim27,Rgcc,Sub1,Sdf2l1,Trem2,Gfra3,Ifitm3,Mgst3,Ifih1,F3,Psmb2,
						Errfi1,Oasl1,Gpnmb,Bhlhe40,Usp18,Kcnj8,Tyrobp,Coro1a,Myo1e,C1qc,Scd1,Lepr,Parp14,Socs5,Ccl5,C1qb,Adrb1,Rtp4,Ddx58,
						Igfbp2,C1qa,Irgm1,Pdia6,Hic1,Ly6e,Dusp18,Blnk,Cdkn3,Alox5ap,Ptpro,Pirb,Fcer1g,Oas1a,Isg15,Emr1,Myo1f,Itgb1,Zak,Prnp,
						Lama2,Cdc25a,Lst1,Gpr146,E2f1,B2m,Cd47,Gbp3,Jun,Sprr2a2,Lgals9,Cd68,Ube2v1,Ppara,Ifi204,Spp1,Snap23,Hmga1,Mpv17,
						Lcp1,Ifi203,Xaf1,Rdh11,Ubtf,Pja2,Ly6a
GO:0051707	response to other organism	29	1050	0.6	1.27E-07	Trim27,Trem2,Ifitm3,Ifih1,Oasl1,Usp18,Kcnj8,Coro1a,C1qc,Scd1,Parp14,Ccl5,C1qb,Rtp4,Ddx58,C1qa,Irgm1,Fcer1g,Oas1a,Isg15,
						Myo1f,B2m,Cd47,Gbp3,Jun,Lgals9,Ifi204,Pja2,Ly6a
GO:0009605	response to external stimulus	40	2021	0.46	3.18E-07	Ppan,Vav1,Trim27,Trem2,Gfra3,Ifitm3,Ifih1,Oasl1,Bhlhe40,Usp18,Kcnj8,Coro1a,C1qc,Scd1,Parp14,Ccl5,C1qb,Adrb1,Rtp4,Ddx58,
						Igfbp2,C1qa,Irgm1,Ptpro,Fcer1g,Oas1a,Isg15,Myo1f,Lama2,B2m,Cd47,Gbp3,Jun,Lgals9,Ppara,Ifi204,Spp1,Rdh11,Pja2,Ly6a
GO:0006950	response to stress	49	2899	0.39	3.97E-07	Gtf2h1,Cdk9,Lpl,Vash1,Trim27,Rgcc,Sdf2l1,Trem2,Ifitm3,Ifih1,F3,Errfi1,Oasl1,Kcnj8,Coro1a,C1qc,Scd1,Parp14,Socs5,Ccl5,C1qb,
						Adrb1,Rtp4,Ddx58,C1qa,Irgm1,Pdia6,Hic1,Cdkn3,Alox5ap,Fcer1g,Oas1a,Isg15,Myo1f,Zak,Prnp,E2f1,B2m,Gbp3,Jun,Ube2v1,Ppara,
						Ifi204,Spp1,Snap23,Hmga1,Mpv17,Lcp1,Pja2
GO:0050789	regulation of biological process	103	9594	0.19	4.68E-07	Cav2,Ebi3,Ppan,Vav1,Gtf2h1,Cdk9,Ctss,Lpl,Lims1,Vash1,Trim27,Rgcc,Sub1,Sdf2l1,Ncbp2,Adamts1,Gzma,Gcm1,Trem2,Psmb8,
						Gfra3,Ifitm3,Mkln1,Ifih1,F3,Zfp593,Errfi1,Oasl1,Gpnmb,Bhlhe40,Usp18,Kcnj8,Tyrobp,Coro1a,Myo1e,C1qc,Scd1,Lepr,L3mbtl3,
						Parp14,Socs5,Ccl5,C1qb,Adrb1,Erf,Ddx58,Pim3,Igfbp2,C1qa,Irgm1,Pdia6,Hic1,Ly6e,Dusp18,Blnk,Rbm15b,Irf2bp2,Fseg,Cdkn3,
						Alox5ap,Ptpro,Pirb,Hsf2,Fcer1g,Oas1a,Isg15,Emr1,Ppp1r3c,Myo1f,Itgb1,Zak,Prnp,AF251705,Lama2,Cdc25a,Lst1,Arrdc3,Gpr146,
						E2f1,Dstn,B2m,Cd47,Jun,Lgals9,Ube2v1,Ppara,Papola,Snx5,Ifi204,Spp1,Snap23,Samd9l,Hmga1,Sox3,Mpv17,Lcp1,Slmap,Irf9,
						Ifi203,Xaf1,Rdh11,Ubtf,Pja2
GO:0006952	defense response	27	1079	0.56	2.09E-06	Trim27,Trem2,Ifitm3,Ifih1,F3,Oasl1,Kcnj8,Coro1a,C1qc,Scd1,Parp14,Ccl5,C1qb,Rtp4,Ddx58,C1qa,Irgm1,Alox5ap,Fcer1g,Oas1a,
						Isg15,Myo1f,B2m,Gbp3,Jun,Snap23,Pja2
GO:0098542	defense response to other organism	22	735	0.64	2.59E-06	Trim27,Trem2,Ifitm3,Ifih1,Oasl1,Kcnj8,Coro1a,C1qc,Scd1,Parp14,Ccl5,C1qb,Rtp4,Ddx58,C1qa,Irgm1,Fcer1g,Oas1a,Isg15,Myo1f,Gbp3,Pja2

Heart samples were obtained from gestational day (GD) 18 C57Bl/6 J fetal mice exposed for 2 weeks prior to conception and during gestation to either 0 ppb (control) or 100 ppb (exposed) sodium (meta) arsenite. After microarray analysis, enriched biological processes were identified based on the 150 differentially expressed (comparing exposed to controls exhibited an unadjusted *p*-value <0.01) genes from the heart using gene ontology (GO) in the STRING database. The specific GO number is reported along with its term description. Each protein from the differentially expressed input that are associated with that biological process are listed in the right column of the table. Data shown represents an n = 3.

**Table 4:** Enriched KEGG pathways in arsenic-exposed GD18 heart tissue.

#Term ID	Term description	Obs gene count	Bkgd gene count	Strength	False discovery rate	Matching proteins in your network (labels)

mmu05020	Prion diseases	5	34	1.33	0.001	C1qc,Ccl5,C1qb,C1qa,Prnp
mmu04380	Osteoclast differentiation	6	122	0.85	0.012	Trem2,Tyrobp,Blnk,Pirb,Jun,Irf9
mmu04662	B cell receptor signaling pathway	5	69	1.02	0.012	Vav1,Cd72,Blnk,Pirb,Jun
mmu05133	Pertussis	5	74	0.99	0.012	C1qc,C1qb,C1qa,Itgb1,Jun
mmu05142	Chagas disease (American trypanosomiasis)	5	101	0.86	0.0254	C1qc,Ccl5,C1qb,C1qa,Jun
mmu04621	NOD-like receptor signaling pathway	6	164	0.72	0.0294	Ccl5,Oas1a,Gbp3,Jun,Ifi204,Irf9
mmu05164	Influenza A	6	165	0.72	0.0294	Ifih1,Ccl5,Ddx58,Oas1a,Jun,Irf9

Heart samples were obtained from gestational day (GD) 18 C57Bl/6 J fetal mice exposed for 2 weeks prior to conception and during gestation to either 0 ppb (control) or 100 ppb (exposed) sodium (meta) arsenite. After microarray analysis, enriched KEGG pathways were identified by STRING based on the 150 differentially expressed (comparing exposed to controls exhibited an unadjusted *p*-value <0.01) genes from the heart. The specific term identification number is reported along with its term description. Each protein from the differentially expressed input that are associated with that pathway are listed in the right column of the table. Data shown represents an n = 3.

**Table 5:** Enriched biological processes (A) and enriched KEGG pathways (B) in arsenic-exposed GD18 lung tissue.

#Term ID	Term description	Obs gene count	Bkgd gene count	Strength	False discovery rate	Matching proteins in your network (labels)

**A.**						
GO:0031497	Chromatin assembly	5	115	1.45	0.0138	Hist1h3g,Hist1h4j,Anp32b,Dnmt3b,Grwd1
GO:0042254	Ribosome biogenesis	6	278	1.15	0.0138	Ddx18,Ddx21,Surf6,C1qbp,Nop56,Rsl1d1
GO:0044085	Cellular component biogenesis	14	2201	0.62	0.0138	Ddx18,Mybl2,Ddx21,Surf6,Vamp8,Rcc2,C1qbp,Hist1h3g,Hist1h4j,
						Nop56,Anp32b,Dnmt3b,Rsl1d1,Grwd1
GO:0006334	Nucleosome assembly	4	78	1.52	0.0162	Hist1h3g,Hist1h4j,Anp32b,Grwd1
**B.**						
mmu00270	Cysteine and methionine metabolism	3	50	1.59	0.0246	Srm,Phgdh,Dnmt3b

Lung samples were obtained from gestational day (GD) 18 C57Bl/6 J fetal mice exposed for 2 weeks prior to conception and during gestation to either 0 ppb (control) or 100 ppb (exposed) sodium (meta) arsenite. After microarray analysis, **(A)** enriched biological processes and **(B)** KEGG pathways were identified based on the 41 differentially expressed (comparing exposed to controls exhibited an unadjusted *p*-value <0.01) genes from the lung using gene ontology (GO) and KEGG terms in the STRING database. The specific GO number or KEGG term ID number is reported along with its term description. Each protein from the differentially expressed input that are associated with that biological process or pathway are listed in the right column of the tables. Data shown represents an n = 3.

**Table 6:** Top 10 enriched cellular components in arsenic-exposed GD18 placenta tissue.

#Term ID	Term description	Obs gene count	Bkgd gene count	Strength	False discovery rate	Matching proteins in your network (labels)

GO:0005622	Intracellular	125	12596	0.13	0.00014	Lhx2,Tollip,Bcap31,Sh3gl1,Cd44,Letm1,Slc30a4,Cdk4,Coro1b,Gstm2,Ndufs2,Capza2,Crk,Calm3,Ubb,Perp,Tbc1d15,Cmpk2_J_ Lgmn,Dek,Ctsr,Samm50,Senp5,Btg3,Mrps23,Psmb8,Rbm22,Thoc3,Ncl,Atf3,Gmps,Snx7,Cpne3,Xpa,Pank4,Ppm1g,Nudt9_J_ Trim24,Lonp2,AI118078,Snx1,Nt5e,Tph1,Palm,Rtp4,Mif,Serpina3g,Ticrr,Wdr74,Ric1,Mcl1,Pim3,Cd3eap,Fanci,Chsy1,Tulp4, Commd3,Irgm1,Bzw1,Rasd1,Zfp207,Xbp1,Zfp445,Tcf25,Gpr107,Kcnb1,Slc4a7,Rcan1,Mtif3,Kcmf1,BC004004,Retsat,Krt7, Rabac1,Zfp706,Deaf1,Arf1,Rpl5,Plekhg5,Hmgb1,Plcxd1,Arfgef1,Htatsf1,Syt6,Dnajc8,Klhl9,Cnep1r1,Tuba1a,Rims1,Igf2bp2, Anapc11,Snrpb,Ifit3,Gbp3,Camk2d,B9d2,Med7,Pkig,Fam110a,Calm1,Ifi204,Hat1,Klhl12,Gmppb,Suv39h1,Wdr45,Ptpn2,Hagh, Pitpna,Srpk1,Setd2,Akap13,Tpd52l2,Zfp637,Phf11d,Tmem55b,Idh3a,Erlin1,Slc3a2,Sec61g,Cnppd1,Tnpo2,Foxp1,Golga5_J_ Entpd4
GO:0005737	Cytoplasm	106	10283	0.15	0.00077	Tollip,Bcap31,Sh3gl1,Cd44,Letm1,Slc30a4,Cdk4,Coro1b,Gstm2,Ndufs2,Capza2,Crk,Calm3,Ubb,Perp,Tbc1d15,Cmpk2,Lgmn_J_ Dek,Ctsr,Samm50,Btg3,Mrps23,Psmb8,Rbm22,Ncl,Gmps,Snx7,Cpne3,Xpa,Pank4,Nudt9,Trim24,Lonp2,AI118078,Snx1,Nt5e_J_ Tph1,Palm,Rtp4,Mif,Serpina3g,Ric1,Mcl1,Pim3,Cd3eap,Fanci,Chsy1,Tulp4,Commd3,Irgm1,Bzw1,Rasd1,Zfp207,Xbp1,Gpr107_J_ Kcnb1,Slc4a7,Rcan1,Mtif3,Kcmf1,BC004004,Krt7,Rabac1,Zfp706,Deaf1,Arf1,Rpl5,Plekhg5,Hmgb1,Plcxd1,Arfgef1,Syt6,Dnajc8, Cnep1r1,Tuba1a,Rims1,Igf2bp2,Anapc11,Snrpb,Ifit3,Gbp3,Camk2d,B9d2,Pkig,Fam110a,Calm1,Ifi204,Klhl12,Gmppb,Wdr45, Ptpn2,Hagh,Pitpna,Srpk1,Akap13,Tpd52l2,Tmem55b,Idh3a,Erlin1,Slc3a2,Sec61g,Tnpo2,Foxp1,Golga5_J_Entpd4
GO:0043227	Membrane-bounded organelle	107	10370	0.15	0.00077	Lhx2,Tollip,Bcap31,Sh3gl1,Cd44,Letm1,Slc30a4,Cdk4,Ndufs2,Calm3,Ubb,Perp,Tbc1d15,Cmpk2,Lgmn,Dek,Ctsr,Samm50_J_ Senp5,Btg3,Mrps23,Psmb8,Rbm22,Thoc3,Ncl,Atf3,Snx7,Cpne3,Xpa,Pank4,Ppm1g,Nudt9,Trim24,Nomo1,Lonp2,Snx1,Nt5e_J_ Palm,Mif,Serpina3g,Ticrr,Wdr74,Ric1,Mcl1,Cd3eap,Fanci,Chsy1,Commd3,Irgm1,Rasd1,Zfp207,Xbp1,Zfp445,Tcf25,Gpr107_J_ Kcnb1,Slc4a7,Rcan1,Mtif3,BC004004,Retsat,Rabac1,Zfp706,Deaf1,Arf1,Rpl5,Plekhg5,Hmgb1,Arfgef1,Htatsf1,Syt6,Dnajc8, Cnep1r1,Tuba1a,Rims1,Igf2bp2,Anapc11,Snrpb,Ifit3,Gbp3,Camk2d,B9d2,Med7,Pkig,Calm1,Ifi204,Hat1,Klhl12,Gmppb_J_ Suv39h1,Ptpn2,Hagh,Srpk1,Setd2,Akap13,Zfp637,Phf11d,Tmem55b,Idh3a,Erlin1,Slc3a2,Sec61g,Cnppd1,Tnpo2,Foxp1,Golga5_J_ Entpd4
GO:0043229	Intracellular organelle	111	11084	0.13	0.00077	Lhx2,Tollip,Bcap31,Sh3gl1,Cd44,Letm1,Slc30a4,Cdk4,Coro1b,Ndufs2,Capza2,Crk,Calm3,Ubb,Perp,Tbc1d15,Cmpk2,Lgmn,Dek_J_ Ctsr,Samm50,Senp5,Btg3,Mrps23,Psmb8,Rbm22,Thoc3,Ncl,Atf3,Snx7,Cpne3,Xpa,Pank4,Ppm1g,Nudt9,Trim24,Lonp2,Snx1_J_ Nt5e,Palm,Mif,Serpina3g,Ticrr,Wdr74,Ric1,Mcl1,Cd3eap,Fanci,Chsy1,Commd3,Irgm1,Rasd1,Zfp207,Xbp1,Zfp445,Tcf25, Gpr107,Kcnb1,Slc4a7,Rcan1,Mtif3,BC004004,Retsat,Krt7,Rabac1,Zfp706,Deaf1,Arf1,Rpl5,Plekhg5,Hmgb1,Arfgef1,Htatsf1, Syt6,Dnajc8,Cnep1r1,Tuba1a,Rims1,Igf2bp2,Anapc11,Snrpb,Ifit3,Gbp3,Camk2d,B9d2,Med7,Pkig,Fam110a,Calm1,Ifi204,Hat1_J_ Klhl12,Gmppb,Suv39h1,Ptpn2,Hagh,Srpk1,Setd2,Akap13,Zfp637,Phf11d,Tmem55b,Idh3a,Erlin1,Slc3a2,Sec61g,Cnppd1_J_ Tnpo2,Foxp1,Golga5,Entpd4
GO:0043231	Intracellular membrane- bounded organelle	100	9507	0.16	0.00077	Lhx2,Tollip,Bcap31,Cd44,Letm1,Slc30a4,Cdk4,Ndufs2,Calm3,Ubb,Perp,Tbc1d15,Cmpk2,Lgmn,Dek,Ctsr,Samm50,Senp5,Btg3_J_ Mrps23,Psmb8,Rbm22,Thoc3,Ncl,Atf3,Cpne3,Xpa,Pank4,Ppm1g,Nudt9,Trim24,Lonp2,Snx1,Nt5e,Palm,Mif,Serpina3g,Ticrr_J_ Wdr74,Ric1,Mcl1,Cd3eap,Fanci,Chsy1,Commd3,Irgm1,Rasd1,Zfp207,Xbp1,Zfp445,Tcf25,Gpr107,Kcnb1,Rcan1,Mtif3, BC004004,Retsat,Rabac1,Zfp706,Deaf1,Arf1,Rpl5,Hmgb1,Arfgef1,Htatsf1,Syt6,Dnajc8,Cnep1r1,Igf2bp2,Anapc11,Snrpb,Ifit3, Gbp3,Camk2d,B9d2,Med7,Pkig,Calm1,Ifi204,Hat1,Klhl12,Gmppb,Suv39h1,Ptpn2,Hagh,Srpk1,Setd2,Akap13,Zfp637,Phf11d, Tmem55b,Idh3a,Erlin1,Slc3a2,Sec61g,Cnppd1,Tnpo2,Foxp1,Golga5_J_Entpd4
GO:0043226	Organelle	113	11550	0.12	0.0015	Lhx2,Tollip,Bcap31,Sh3gl1,Cd44,Letm1,Slc30a4,Cdk4,Coro1b,Ndufs2,Capza2,Crk,Calm3,Ubb,Perp,Tbc1d15,Cmpk2,Lgmn,Dek_J_ Ctsr,Samm50,Senp5,Btg3,Mrps23,Psmb8,Rbm22,Thoc3,Ncl,Atf3,Snx7,Cpne3,Xpa,Pank4,Ppm1g,Nudt9,Trim24,Nomo1,Lonp2_J_ Snx1,Nt5e,Palm,Mif,Serpina3g,Ticrr,Guca2b,Wdr74,Ric1,Mcl1,Cd3eap,Fanci,Chsy1,Commd3,Irgm1,Rasd1,Zfp207,Xbp1_J_ Zfp445,Tcf25,Gpr107,Kcnb1,Slc4a7,Rcan1,Mtif3,BC004004,Retsat,Krt7,Rabac1,Zfp706,Deaf1,Arf1,Rpl5,Plekhg5,Hmgb1, Arfgef1,Htatsf1,Syt6,Dnajc8,Cnep1r1,Tuba1a,Rims1,Igf2bp2,Anapc11,Snrpb,Ifit3,Gbp3,Camk2d,B9d2,Med7,Pkig,Fam110a_J_ Calm1,Ifi204,Hat1,Klhl12,Gmppb,Suv39h1,Ptpn2,Hagh,Srpk1,Setd2,Akap13,Zfp637,Phf11d,Tmem55b,Idh3a,Erlin1,Slc3a2, Sec61g_J_Cnppd1,Tnpo2,Foxp1,Golga5_J_Entpd4
GO:0005634	Nucleus	72	6330	0.19	0.0039	Lhx2,Tollip,Cd44,Cdk4,Ndufs2,Calm3,Ubb,Dek,Senp5,Btg3,Mrps23,Psmb8,Rbm22,Thoc3,Ncl,Atf3,Cpne3,Xpa,Pank4,Ppm1g_J_ Nudt9,Trim24,Lonp2,Nt5e,Palm,Mif,Serpina3g,Ticrr,Wdr74,Mcl1,Cd3eap,Fanci,Commd3,Rasd1,Zfp207,Xbp1,Zfp445,Tcf25, Gpr107,Rcan1,BC004004,Retsat,Zfp706,Deaf1,Rpl5,Hmgb1,Arfgef1,Htatsf1,Dnajc8,Cnep1r1,Igf2bp2,Anapc11,Snrpb,Gbp3_J_ Camk2d,B9d2,Med7,Pkig,Calm1,Ifi204,Hat1,Suv39h1,Ptpn2,Srpk1,Setd2,Akap13,Zfp637,Phf11d,Slc3a2,Cnppd1,Tnpo2,Foxp1
GO:0110165	Cellular anatomical entity	138	15632	0.08	0.0039	Lhx2,Tollip,Bcap31,Sh3gl1,Cd44,Letm1,Slc30a4,Cdk4,Coro1b,Gstm2,Ndufs2,Capza2,Crk,Calm3,Ubb,Perp,Tbc1d15,Cmpk2, Lgmn,Dek,Ctsr,Samm50,Senp5,Btg3,Mrps23,Psmb8,Rbm22,Thoc3,Ncl,Atf3,Cd82,Gmps,Snx7,Cpne3,Xpa,Pank4,Ppm1g,Nudt9_J_ Trim24,Lyve1,Nomo1,Lonp2,AI118078,Snx1,Nt5e,Tph1,Palm,Rtp4,Mif,Serpina3g,Ticrr,Guca2b,Wdr74,Ric1,Mcl1,Pim3, Cd3eap,Fanci,Tmem183a,Chsy1,Tulp4,Commd3,Irgm1,Bzw1,Rasd1,Zfp207,Xbp1,Zfp445,Tcf25,Gpr107,Tmem104,Kcnb1_J_ Slc4a7,Rcan1,Mtif3,Kcmf1,BC004004,Cadm4,Retsat,Krt7,Tmem140,Rabac1,Zfp706,Deaf1,Arf1,Prl3d1,Rpl5,Plekhg5,Hmgb1, Plcxd1,Arfgef1,Htatsf1,Syt6,Ptprb,Dnajc8,Klhl9,Cnep1r1,Tuba1a,Rims1,Igf2bp2,Anapc11,Snrpb,Ifit3,Igf2,Gbp3,Camk2d,B9d2, Med7,Pkig,Fam110a,Calm1,Ifi204,Hat1,Klhl12,Kcna6,Gmppb,Suv39h1,Wdr45,Ptpn2,Hagh,Pitpna,Srpk1,Setd2,Akap13_J_ Tpd52l2,Zfp637,Phf11d,Tmem55b,Idh3a,Erlin1,Slc3a2,Smim19,Sec61g,Cnppd1,Tnpo2,Foxp1,Golga5_J_Entpd4
G0:0005654	Nucleoplasm	43	3261	0.25	0.0148	Lhx2,Tollip,Cdk4,Ndufs2,Calm3,Ubb,Dek,Psmb8,Rbm22,Thoc3,Ncl,Atf3,Cpne3,Xpa,Ppmlg,Nudt9,Trim24,Nt5e,Palm,Mif,Ticrr, Wdr74,Mell,Cd3eap,Fanci,Zfp207,Gprl07,Deafl,Rpl5,Arfgefl,Htatsfl,Dnajc8,Anapcll,Snrpb,Med7,Caimi,Ifi204,Hatl, Suv39hl,Ptpn2,Srpkl,Phflld,Slc3a2
G0:0031981	Nuclear lumen	46	3771	0.22	0.0439	Lhx2,Tollip,Cdk4,Ndufs2,Calm3,Ubb,Dek,Senp5,Psmb8,Rbm22,Thoc3,Ncl,Atf3,Cpne3,Xpa,Ppmlg,Nudt9,Trim24,Nt5e,Palm, Mif,Ticrr,Wdr74, Mell, Cd3eap, Fanci, Zfp207,Gprl07,BC004004,Deafl,Rpl5,Hmgbl,Arfgefl,Htatsfl,Dnajc8,Anapcll,Snrpb, Med7, Caimi, Ifi204,Hatl,Suv39hl,Ptpn2,Srpkl,Phflld,Slc3a2

Placenta samples were obtained from gestational day (GD) 18 C57B1/6 J fetal mice exposed for 2 weeks prior to conception and during gestation to either 0 ppb (control) or 100 ppb (exposed) sodium (meta) arsenite. After microarray analysis, enriched cellular components were identified based on the 165 differentially expressed (comparing exposed to controls exhibited an unadjusted p-value <0.01) genes from the placenta using gene ontology (GO) in the STRING database. The specific GO number is reported along with its term description. Each protein from the differentially expressed input that are associated with that biological process are listed in the right column of the table. Data shown represents an n = 3.

## Data Availability

Data will be made available on request.

## Appendix A. Supporting information

Supplementary data associated with this article can be found in the online version at doi:10.1016/j.tox.2026.154420.
