# Supplementary material for: Prenatal arsenic exposure and gene expression in fetal liver, heart, lung, and placenta
Source: Toxicology. Author manuscript; Available in PMC 2026 Apr 20. (PMC13094789; doi:10.1016/j.tox.2026.154420)
Supplement: MMC2 [file NIHMS2151723-supplement-MMC2.docx]

**Supplemental Data**

**Title:** Prenatal Arsenic Exposure and Gene Expression in Fetal Liver, Heart, Lung, and Placenta

Author names & affiliations:

K.A. Rychlik^1,2^, S. Sanchez^1^, C. Kashiwagi^1^, J. Liao^1^, A. Mathur^1^, E.J. Illingworth^1^, A. Kleensang^1^, A. Maertens^1^, F.C.M. Sillé^1^

^1^ Department of Environmental Health and Engineering, Bloomberg School of Public Health, Johns Hopkins University, Baltimore, MD, USA

^2^ Public Health Program, School of Health Professions, Mayborn College of Health Sciences, University of Mary Hardin-Baylor, Belton, TX, USA

Corresponding author: F.C.M. Sillé, PhD, Johns Hopkins Bloomberg School of Public Health, 615 N. Wolfe st. RM E7628, Baltimore, MD 21205; email: [fsille1@jhu.edu](mailto:fsille1@jhu.edu)

**Keywords**: arsenic, prenatal, mRNA, microarray, immune

**Running Head:** Prenatal Arsenic: Gene Expression in Multiple Fetal Tissues

**Supplemental Figures**


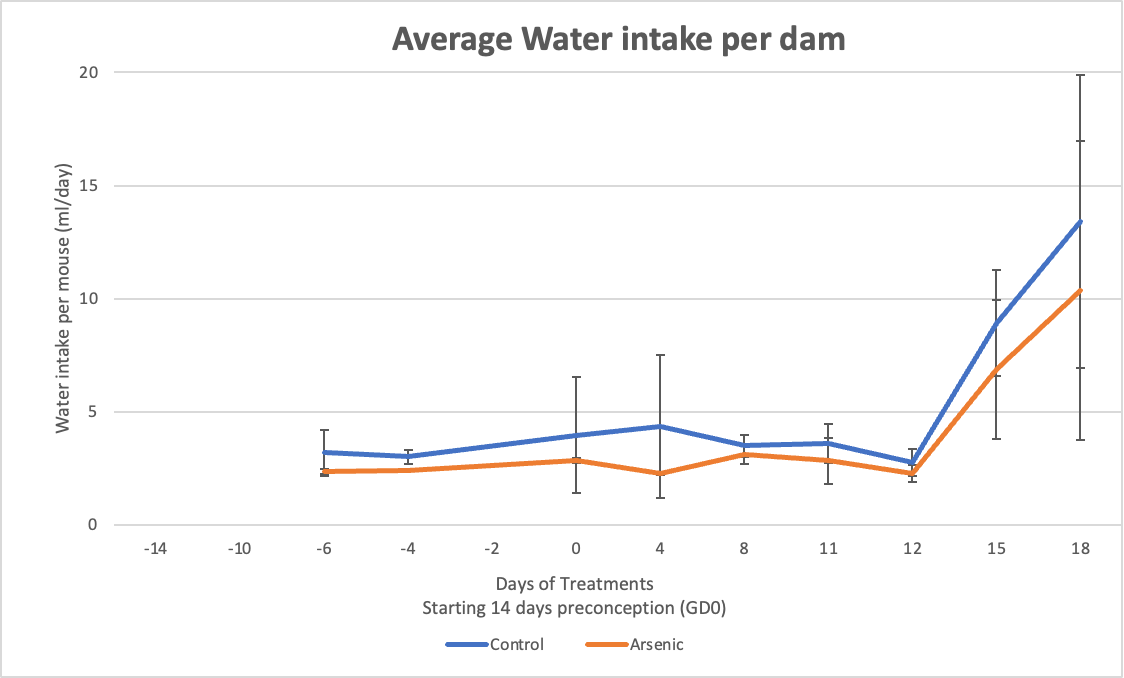


*

**Supplemental Figure S1.** Average water intake per dam per day. From two weeks prior to mating until tissue collection at GD18, mice were exposed to 0 (“Control”) or 100 ppb sodium (meta) arsenite (“Arsenic”) in drinking water, *ad libitum*. Female mice were housed in groups of five prior to mating, after which they were housed individually in order to accurately monitor food and water intake. Overall, no significant differences in water consumption were noted between exposure groups, except for a minor significant difference at 4 days prior to mating. * *P*<0.05


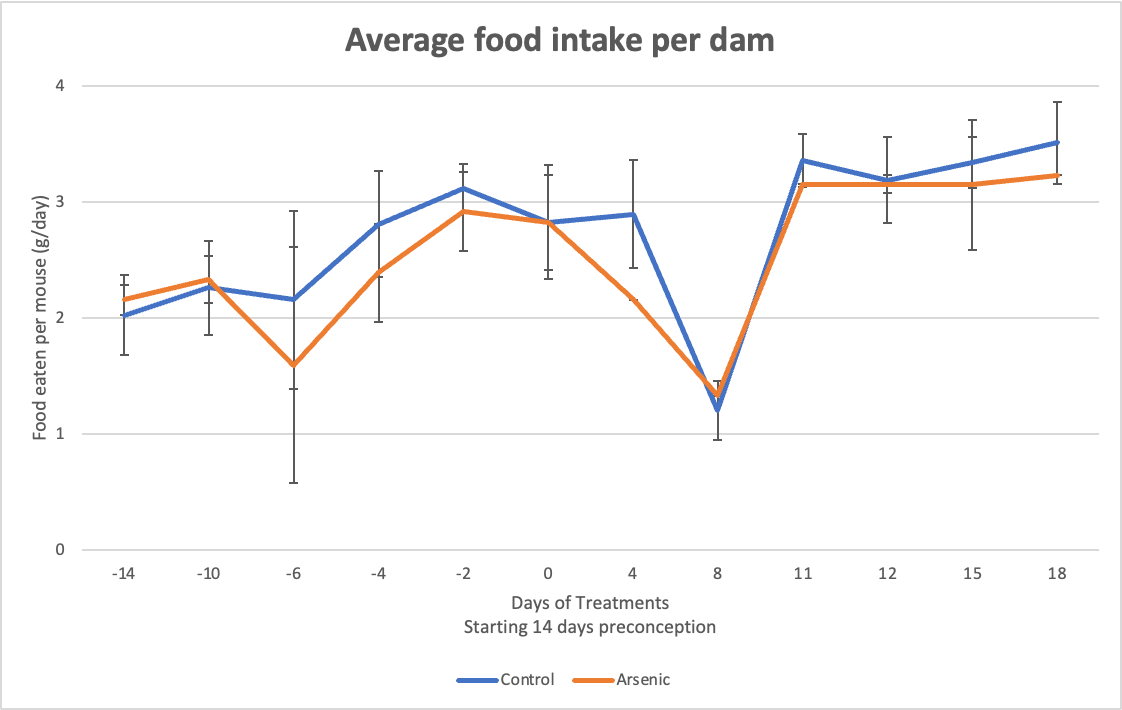


**Supplemental Figure S2.** Average food intake per dam per day. From two weeks prior to mating until tissue collection at GD18, mice were exposed to 0 (“Control”) or 100 ppb sodium (meta) arsenite (“Arsenic”) in drinking water, *ad libitum*. Throughout the experiment mice were fed low arsenic chow diet (AIN-93M, Research Diets, Inc.), *ad libitum*. Female mice were housed in groups of five prior to mating, after which they were housed individually in order to accurately monitor food and water intake. Overall, no significant differences in water consumption were noted between exposure groups, except for a minor significant difference at 4 days prior to mating. * *P*<0.05


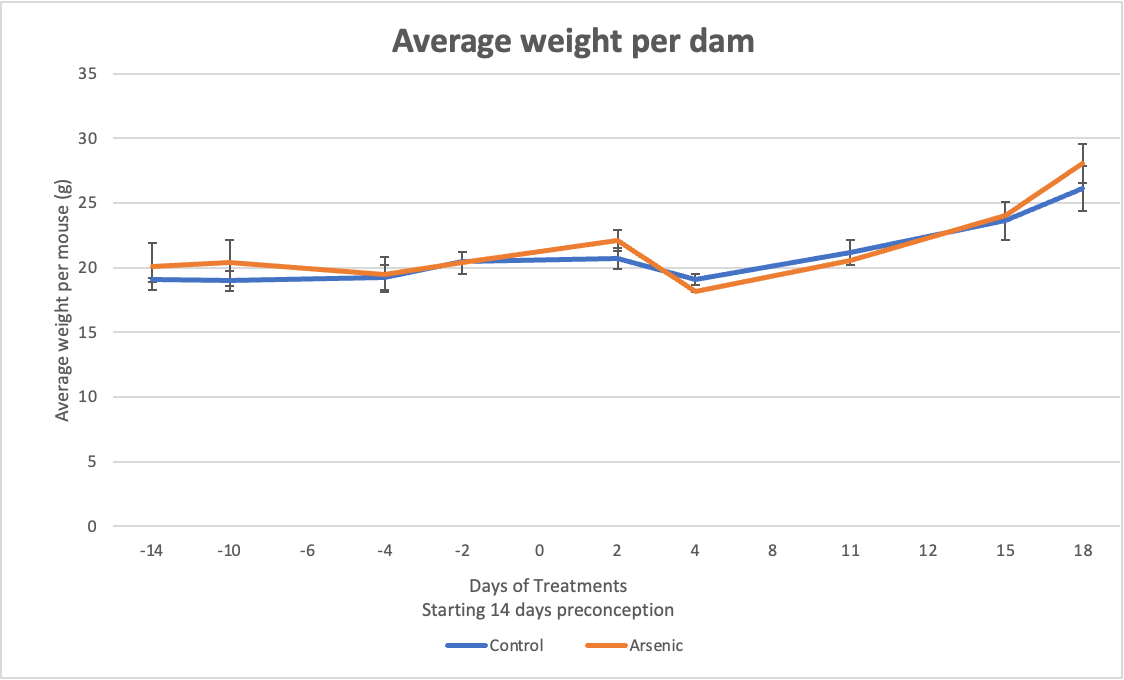


**Supplemental Figure S3.** Average weight per dam per day. From two weeks prior to mating until tissue collection at GD18, mice were exposed to 0 (“Control”) or 100 ppb sodium (meta) arsenite (“Arsenic”) in drinking water, *ad libitum*. Overall, no significant changes in average dam weight was observed throughout the experiment.

**Supplemental Figure S4.** Arsenic effect on glucocorticoid receptor pathway expression. From two weeks prior to mating until tissue collection at GD18, mice were exposed to 0 (“Control”) or 100 ppb sodium (meta) arsenite (“Arsenic”) in drinking water, *ad libitum*. When querying potential impact of prenatal arsenic exposure on the glucocorticoid receptor pathway, 9 (*Aqp1, H6pd, Cyb561, Zhx3, Slc22a5, Pdgfrb, Sgk1, Spsb1*, and *Aff1*) out of 12 previously identified genes associated with arsenic exposure (Meakin et al. 2019) were dysregulated in fetal liver, heart, lung, and placenta tissues

**Supplemental Tables**

**Table S1. RTqPCR sample numbers.** For the validation by RT-qPCR fetal tissue samples, 2 different exposure groups (unexposed vs. 100 ppb inorganic arsenic exposed) * 4 different tissues (fetal heart, liver, lung and placenta) * 2-15 animals per exposure group were used.

**Table S2. Complete List of Differential Gene Expression Analysis for Heart Tissue.** Heart samples were obtained from gestational day (GD) 18 C57Bl/6J fetal mice exposed for 2 weeks prior to conception and during gestation to either 0 ppb (control) or 100 ppb (exposed) sodium (meta) arsenite. After microarray analysis, the expression of all genes from heart tissue were compared for arsenic exposed to unexposed controls. Data shown represents an n=3.

**Table S3. Complete List of Differential Gene Expression Analysis for Liver Tissue.** Liver samples were obtained from gestational day (GD) 18 C57Bl/6J fetal mice exposed for 2 weeks prior to conception and during gestation to either 0 ppb (control) or 100 ppb (exposed) sodium (meta) arsenite. After microarray analysis, the expression of all genes from liver tissue were compared for arsenic exposed to unexposed controls. Data shown represents an n=3.

**Table S4. Complete List of Differential Gene Expression Analysis for Lung Tissue.** Lung samples were obtained from gestational day (GD) 18 C57Bl/6J fetal mice exposed for 2 weeks prior to conception and during gestation to either 0 ppb (control) or 100 ppb (exposed) sodium (meta) arsenite. After microarray analysis, the expression of all genes from lung tissue were compared for arsenic exposed to unexposed controls. Data shown represents an n=3.

**Table S5. Complete List of Differential Gene Expression Analysis for Placenta Tissue.** Placenta samples were obtained from gestational day (GD) 18 C57Bl/6J fetal mice exposed for 2 weeks prior to conception and during gestation to either 0 ppb (control) or 100 ppb (exposed) sodium (meta) arsenite. After microarray analysis, the expression of all genes from placenta tissue were compared for arsenic exposed to unexposed controls. Data shown represents an n=3.

**Table S6. Arsenic effect on glucocorticoid receptor pathway expression.** From two weeks prior to mating until tissue collection at GD18, mice were exposed to 0 (“Control”) or 100 ppb sodium (meta) arsenite (“Arsenic”) in drinking water, *ad libitum*. When querying potential impact of prenatal arsenic exposure on the glucocorticoid receptor pathway, 9 (*Aqp1, H6pd, Cyb561, Zhx3, Slc22a5, Pdgfrb, Sgk1, Spsb1*, and *Aff1*) out of 12 previously identified genes associated with arsenic exposure (Meakin et al. 2019) were dysregulated in fetal liver, heart, lung, and placenta tissues.

**Table S7. Complete List of Enriched Biological Processes in arsenic-exposed GD18 Liver Tissue.** Liver samples were obtained from gestational day (GD) 18 C57Bl/6J fetal mice exposed for 2 weeks prior to conception and during gestation to either 0 ppb (control) or 100 ppb (exposed) sodium (meta) arsenite. After microarray analysis, enriched biological processes were identified in STRING based on the 240 differentially expressed (comparing exposed to controls exhibited an unadjusted *p*-value <0.01) genes from the liver using gene ontology (GO). The specific GO number is reported along with its term description. Each protein from the differentially expressed input that are associated with that biological process are listed in the right column of the table. Data shown represents an n=3.
